# Developmental Dynamics of Bioactive Composition and Biological Activities of Methanolic Extracts from Orange (*Citrus sinensis*) Peel: Comprehensive Metabolite Profiling, Antioxidant Capacity, and Antibacterial Activity

**DOI:** 10.3390/plants15193017

**Published:** 2026-10-01

**Authors:** Sercan Önder

**Affiliations:** Department of Agricultural Biotechnology, Faculty of Agriculture, Isparta University of Applied Sciences, Isparta 32200, Türkiye; sercanonder@isparta.edu.tr

**Keywords:** sweet orange, fruit development, phenolic compounds, flavonoids, organic acids, mineral composition, antimicrobial activity

## Abstract

Although the chemical composition and biological properties of orange peels undergo significant changes during fruit development, the extent of variation in metabolites and their activities remains poorly characterized. In this study, the fruit development characteristics, chemical composition, antioxidant capacity and antibacterial activities of orange peel extracts were evaluated across six sampling times corresponding to 120 to 250 days after flowering (DAF) using the Valencia cultivar. All pomological variables increased during fruit development except peel thickness and fruit juice pH. Both total phenolics and flavonoid content and antioxidant capacity were higher at early stages and their content and activity decreased as fruit development progressed. Of the 58 compounds (5 carotenoids, 19 flavonoids, 10 phenolic acids, 9 organic acids, 9 vitamins, 6 aroma-volatile compounds), 38 were quantitatively determined, with *β*-carotene, hesperidin, and ferulic acid being predominant compounds among carotenoids, flavonoids, and phenolic acids, respectively. Generally, the amount of individual carotenoids, flavonoids and phenolic acid content increased with fruit development, except epicatechin and syringic acid. Citric and malic acids were the predominant organic acids, while ascorbic acid was the major water-soluble vitamin, reaching a maximum concentration of 1508.3 mg kg^−1^ in Stage V. Among the volatile compounds, limonene was predominant. Mineral content of peels decreased steadily during fruit development and Ca, K and Mg were predominant elements. Antibacterial activity varied with developmental stage and microorganism; *Bacillus cereus* was inhibited by extracts from all stages, while the largest inhibition zone (27.67 mm) was observed against *Enterococcus faecalis* with the Stage II extract. Multivariate analysis further distinguished the developmental stages based on their metabolite profiles, with the first two principal components explaining 84.63% of the total variance. Overall, the chemical and biological properties of Valencia orange peel exhibited dynamic and compound-specific changes throughout development, highlighting the importance of developmental stage when evaluating the potential use of orange peel.

## 1. Introduction

Citrus species (*Rutaceae*) fruits are among the most widely cultivated and consumed fruits due to their health benefits, high nutritional value, desirable sensory characteristics, and relatively low cost. Citrus crops encompass approximately 140 genera and 1300 species, and the presence of such diversity contributes to substantial differences in fruit types, nutritional composition, sensory characteristics, and bioactive components of these fruits [1]. Global citrus production reached 165,627,078.38 tons in 2024, of which 67,244,550.08 tons (40.60%) were oranges (*Citrus sinensis* L.) [2]. Türkiye ranks thirteenth in global orange production and cultivates various varieties, particularly Washington Navel, Valencia and Blood Orange. Türkiye’s orange production reached 1,842,044.99 tons in 2024, of which 232,044.99 tons were exported [2]. Orange cultivation is particularly concentrated in the Mediterranean Region of Türkiye.

The production and processing of orange fruits generate large amounts of by-products, such as peel, pulp and seeds [3]. Orange peel may constitute approximately 44% of the fruit’s weight and comprises the main part of orange by-products [4]. However, orange peel is mostly considered a waste material, which is mostly discarded and thereby contributing to environmental pollution [5] or used for low-value purposes, such as cattle feed [6]. Nevertheless, orange peel is rich in phenolics, flavonoids, vitamins, minerals and essential oils; therefore, it has considerable potential as a source of natural antioxidants and functional ingredients [4,6,7]. Globally, citrus-derived industrial waste is estimated to exceed 40 million tons annually, and considerable research effort has been directed toward converting citrus waste into value-added materials to mitigate environmental pollution and associated damage [8]. Therefore, identifying constituents of orange peel is important not only for obtaining high-value ingredients for food and nutraceuticals but also for reducing waste.

Citrus peel consists of two main anatomical regions: the outer flavedo (epicarp) and the underlying albedo (mesocarp). The flavedo has a dense cellular structure and contains oil glands where essential oils are stored, whereas the albedo exhibits a looser and more spongy tissue structure [4]. These structural differences may also affect the distribution of bioactive compounds within different layers of the peel. Indeed, the distribution of phenolic compounds within the peel of orange has been reported to vary depending on both location and developmental stage of fruits, with flavedo being richer than albedo for some phenolic acids, flavanols, flavones, and particularly polymethoxyflavonoids [9]. In contrast, glycosylated flavanones have been reported to occur at higher levels in albedo [7].

The biological properties of orange peel are largely associated with phenolic and other bioactive compounds. Total phenolic and flavonoid content and antioxidant capacities of orange peel extracts with different solvents vary depending on the extraction conditions, which affect the composition and quantity of phenolic acids within the extracts [4]. Sweet orange peel extract was found to exhibit high antioxidant capacity, which may be used to improve the oxidative stability of vegetable oils. The same study reported that citrus peel extracts had antimicrobial activities against both Gram-positive and Gram-negative bacteria as well as some fungi [8]. These findings demonstrate that orange peel is an important plant resource that should be evaluated not only in terms of its chemical composition but also for its biological activities.

However, the amounts of bioactive components in orange peel are not constant throughout fruit development and ripening. A study of different sweet orange varieties showed that total phenolic and flavonoid content and antioxidant capacity varied depending on the developmental stage, with some compounds occurring at higher levels, particularly during the early stages of development [10]. Several phenolic compounds occurred at higher levels during immature and semi-mature stages and decreased at the commercial maturity stage in orange [7]. Total phenolic and flavonoid contents and antioxidant activity generally decreased during fruit ripening and a significant proportion of phenolic acids exhibited a similar pattern in navel orange as well [11]. These findings indicate that the developmental stage of fruit is an important factor to consider when evaluating chemical and biological properties of orange peel.

Changes associated with fruit development are not limited to phenolic compounds. Mineral levels have also been reported to change dynamically throughout fruit development in sweet orange varieties, with peel accumulating some mineral elements at higher concentrations than fruit flesh [12]. Similarly, the composition of orange peel essential oils has been shown to change significantly throughout fruit development, with the quantity of volatile compounds fluctuating in connection with development stages [13]. Thus, evaluating the biological potential of orange peel based on a single group of metabolites may be insufficient for a better understanding of metabolite changes that occur during fruit development.

Although there are studies investigating the antimicrobial potential of orange peel, most of these studies focused on the activity of specific extracts or essential oils obtained at a single developmental or harvesting stage. For example, citrus peel extracts have been shown to exhibit antimicrobial activity against various foodborne microorganisms [8]. In addition, molecular docking analyses of *D*- and *L*-limonene, which are major components of citrus peel essential oils, revealed that these compounds may exhibit a more favorable binding affinity toward the antifungal target CYP51 than toward the antibacterial target GyrB [1]. However, information on how the chemical composition of sweet orange peel and its effects on antibacterial activity across successive developmental stages remains limited.

Although several reports demonstrated developmental changes in specific groups of components, such as phenolic compounds, antioxidant capacity, volatile compounds and mineral elements, in citrus fruits [7,10,11,12,13], detailed studies evaluating the developmental stage-dependent changes in other compounds, such as organic acids, vitamins and mineral elements in orange peel along with their antioxidant capacity and antibacterial activity, remain limited. Therefore, better characterization of the chemical composition and associated biological properties of orange peel at different developmental stages is important for understanding developmental metabolite dynamics and assessing the potential of peels for the development of value-added applications.

Thus, this study aimed to determine the effect of fruit developmental stage on the chemical composition and biological potential of orange peel and to evaluate the potential of peels for functional food and nutraceutical applications. In this study, changes in the bioactive composition of peel samples obtained from six consecutive developmental stages of orange fruits were evaluated. The antioxidant capacity and antibacterial activities of the extracts against Gram-positive and Gram-negative bacteria were determined, while polyphenols, organic acids, vitamins and elemental composition were identified and quantified at the same time.

## 2. Materials and Methods

### 2.1. Plant Materials

Orange variety “Valencia” fruits were used and fruits sampled from a farmers’ orchard in Serik district, Antalya province, Türkiye. Standard cultivation practices were followed in the orchard. Fruit development and phenological characteristics were monitored at regular intervals from the flowering period onward. The average monthly temperature, total precipitation and relative humidity recorded in the study area during 2024, together with the corresponding long-term averages for 1972–2024, were obtained from the General Directorate of Meteorology and presented in Table 1.

Fruits were sampled at six intervals during 2024, corresponding to different fruit development stages (Table 2). Developmental stages were defined according to the number of days after flowering (DAF). The flowering date was designated as Apr 29, 2024, when all bloomed flowers withered, and the sampling stages corresponded to 120, 150, 180, 210, 230, and 250 DAF, respectively. The orchard was monitored bi-weekly throughout the growing season, and sampling dates were determined according to the observed developmental progression of the fruits and the approach of the commercial harvest period. These sampling points covered the progression of fruit development from the early green stage through the onset of peel coloration and subsequent ripening.

**Figure 1:**
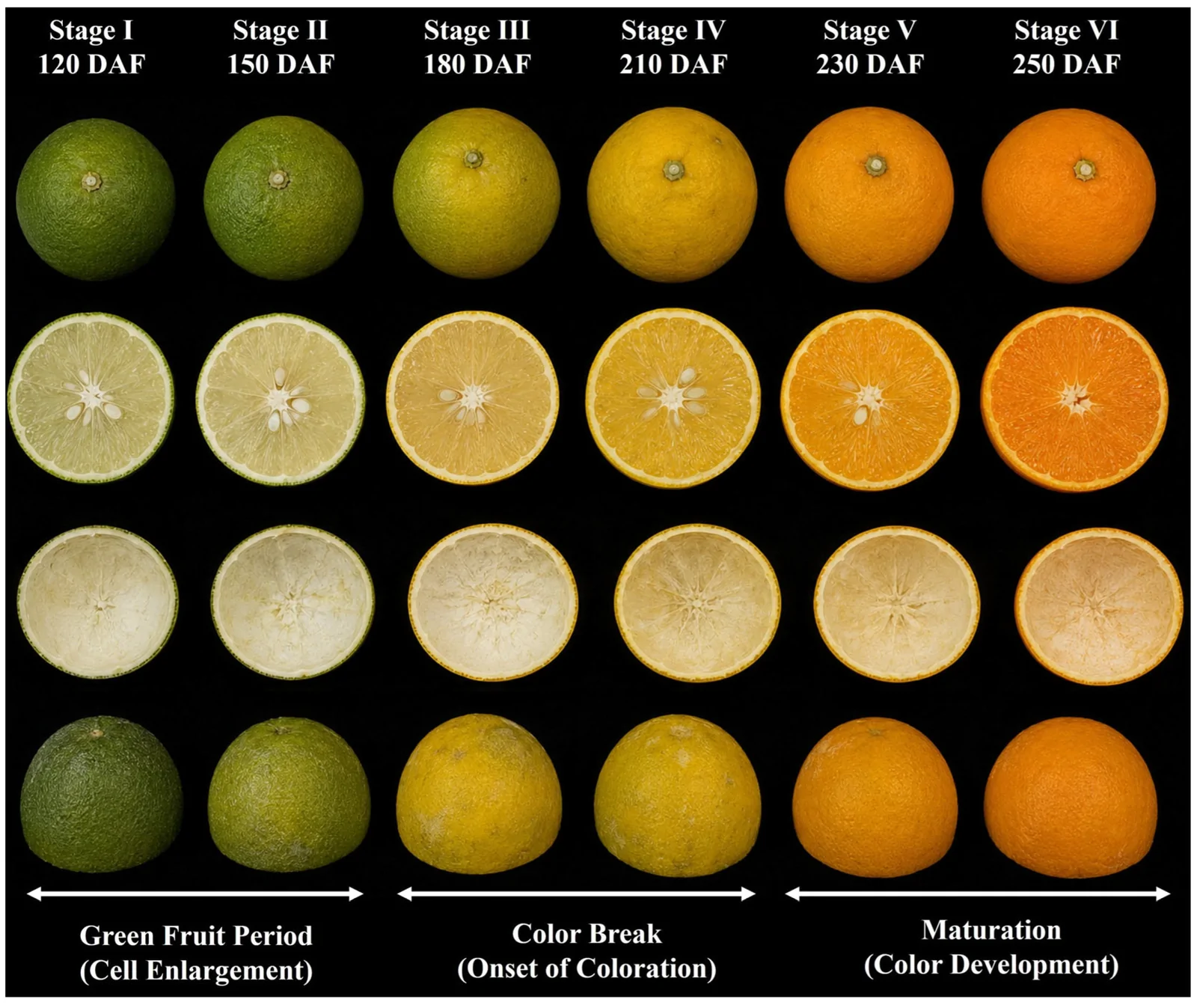
Morphological and developmental changes in Valencia orange (*C. sinensis*) fruits across six sampling times corresponding to different developmental stages from 120 to 250 DAF.

A total of 15 trees were used in the study and assigned to three biological replicates, with five trees per replicate. All 15 trees were located within the same orchard plot and were selected from a relatively uniform area and from trees to minimize potential variation due to differential growth and within-location variation of fruits among the sampled trees. At each developmental stage, two fruits were collected from each of the five trees in each replicate, resulting in 10 fruits per replicate and a total of 30 fruits per developmental stage. Fruits were randomly hand-picked from different areas of the tree canopy, and only fruits free from visible physical damage or disease symptoms were selected. The harvested fruits were transported to the laboratory under cold-chain conditions within 3 h, after which pomological analyses were performed (Figure 1).

### 2.2. Pomological Characteristics

The fruits were washed with distilled water to remove dust and other surface contaminants, then dried with paper towels. The fruits were subsequently divided into appropriate subsamples for pomological and biochemical analyses. Fruit length (mm) and width (mm) were measured using an electronic caliper. These measurements were performed on all 30 fruits sampled at each developmental stage.

The fresh weight (g) of each fruit was determined individually with a precision balance. Following removal of fruit flesh, the thickness (mm) of the remaining peel was measured using an electronic caliper, and the peel weight (g) was determined individually with a precision balance. Peel thickness and peel weight were measured for 15 fruits at each developmental stage. Fruit pulp samples were collected separately from the same 15 fruits, dried in an oven at 80 °C for 120 h, and then weighed to determine the dry weight (g) of the fruit pulp.

To determine the soluble solids content (SSC), orange juice was extracted using a manual fruit press and filtered through coarse filter paper. A few drops of the filtered juice were transferred to a digital refractometer (Hanna HI 96801), and the SSC was expressed as °Brix (%).

The pH value of fruit juices obtained at each developmental stage was measured using a pH meter (Milwaukee MW 150). The same juice samples used for SSC determination were used for pH measurement.

### 2.3. Extraction and Lyophilization of Orange Peels

After the pomological measurements, the remaining fruits were cut in half using a stainless-steel blade, and the pulp and juice were removed using a manual juicer. Orange peels containing the flavedo and albedo layers were separated according to developmental stages and dried at 40 °C for 48 h. The dried peel samples were then ground into a fine powder using liquid nitrogen. For each developmental stage, 20 g of the powdered peel samples were weighed and transferred into 200 mL of 70% (*v*/*v*) methanol. The extraction was performed at 45 °C for 1 h, followed by incubation in a shaking incubator (150 rpm) at room temperature for 5 h. After incubation, the extracts were filtered under vacuum through Whatman No. 4 filter paper to remove peel particles. Methanol was removed from the resulting filtrates using a rotary evaporator (IKA RV 10 Digital Rotary Evaporator, IKA, Staufen, Germany). To shorten the lyophilization time, the methanol-free extracts were first frozen at −20 °C for 24 h and subsequently lyophilized for 36 h at −55 °C and 0.001 bar using a Labconco FreeZone 6 Liter Freeze Dry System (Labconco, Kansas City, MO, USA). The lyophilization step removed the remaining water from extractions, yielding dry powdered orange peel extracts suitable for long-term storage.

### 2.4. Biochemical Analyses

A 0.25 g portion of each lyophilized extract was weighed and dissolved in 10 mL of cold 70% (*v*/*v*) methanol. Total phenolics and total flavonoid content as well as antioxidant capacity were determined from these extracts. CUPRAC (cupric ion reducing antioxidant capacity) and DPPH (2,2-diphenyl-1-picrylhydrazyl) assays were used to evaluate antioxidant capacity. Total phenolics content was determined using the Folin–Ciocalteu method [14]. Briefly, 100 µL of the methanolic extract was mixed with 2.5 mL of distilled water and 100 µL of Folin–Ciocalteu reagent. After incubation in the dark for 6 min, 500 µL of 20% sodium carbonate solution was added. The samples were then incubated in the dark for 30 min to allow color development, and the absorbance was measured at 760 nm using a spectrophotometer (Shimadzu UV-1280, Kyoto, Japan). Total phenolic content was calculated using a gallic acid calibration curve and expressed as mg gallic acid equivalents per g of dry weight (mg GAE g^−1^ DW).

Total flavonoid content was determined using the aluminum chloride method [15]. Briefly, 250 µL of the methanolic extract was mixed with 1.25 mL of distilled water and 75 µL of 5% sodium nitrite solution. After incubation in the dark for 6 min, 150 µL of 10% aluminum chloride solution was added. The mixture was incubated in the dark for an additional 5 min, followed by the addition of 500 µL of 1 M sodium hydroxide and 275 µL of distilled water. After incubation in the dark for 15 min, the absorbance was measured at 510 nm. Total flavonoid content was calculated using a catechin calibration curve and expressed as mg catechin equivalents per g of dry weight (mg catechin g^−1^ DW).

Antioxidant capacity was determined with CUPRAC and DPPH assays. For CUPRAC assay, 1 mL of copper(II) solution (10 mM), 1 mL of neocuproin solution (7.5 mM) and 1 mL of ammonium acetate buffer (1 M, pH 7.0) were added to the test tubes [16]. Subsequently, 0.6 mL of the methanolic extract and 0.5 mL of distilled water were added to the reaction mixture to obtain a final volume of 4.1 mL. The reaction mixture was incubated at room temperature for 30 min, after which the absorbance was measured at 450 nm against a reagent blank without the extract. CUPRAC activity was expressed as µmol Trolox equivalents per g of dry weight (µmol Trolox g^−1^ DW).

DPPH radical scavenging capacity was determined using the stable radical 2,2-diphenyl-1-picrylhydrazyl (DPPH) [17]. Briefly, 1 mL of the methanolic extract, 1 mL of 99% ethanol and 2 mL of DPPH solution (0.2 mM) were mixed in test tubes. The reaction mixture was incubated in the dark for 30 min, and the absorbance was measured at 515 nm against ethanol as the reference. DPPH radical scavenging capacity was expressed as µmol Trolox equivalent per g of dry weight (µmol Trolox g^−1^ DW).

### 2.5. Sample Preparation for Metabolite Analysis

For the extraction of phenolic, flavonoid and carotenoid components, 0.5 g of the lyophilized methanolic extract was resolved in 10 mL of methanol/acetonitrile (50:50, *v*/*v*) containing 0.1% (*v*/*v*) formic acid. The resulting mixture was vortexed for 5 min and then centrifuged at 4000 rpm for 15 min at 4 °C. The supernatant was filtered through a 0.22 μm PTFE membrane filter and transferred to vials for UHPLC-MS/MS analysis.

For the extraction of organic acids, 0.5 g of the lyophilized methanolic extract was resolved in 10 mL of methanol:water (20:80, *v*/*v*) containing 0.1% (*v*/*v*) formic acid. The resulting mixture was vortexed for 5 min and centrifuged at 4000 rpm for 15 min at 4 °C. The supernatant was filtered through a 0.22 μm PTFE membrane filter and transferred to vials for UHPLC-MS/MS analysis.

For the extraction of water-soluble vitamins, 0.5 g of the lyophilized methanolic extract was resolved in 10 mL of methanol:water (50:50, *v*/*v*). Then the samples were homogenized at 10,000 rpm for 5 min using an Ultra-Turrax T25 homogenizer. The resulting homogenates were centrifuged at 4000 rpm for 15 min at 4 °C. The supernatant was filtered through a 0.22 μm PTFE membrane filter and transferred to vials for UHPLC-MS/MS analysis.

### 2.6. UHPLC-MS/MS Analyses

Ultra-high-performance liquid chromatography-tandem mass spectrometry (UHPLC-MS/MS) was used to profile phenolic compounds, flavonoids, carotenoids, organic acids and water-soluble vitamins in the methanolic extracts obtained from different developmental stages of orange peel. The analyzed phenolic compounds were ferulic, rosmarinic, caffeic, caftaric, *p*-coumaric, chlorogenic, gallic, syringic, ellagic and vanillic acids. The flavonoid compounds were cyanidin, delphinidin, apigenin, apigenin-7-glucoside, hesperidin, naringin, narirutin, catechin, epicatechin, epicatechin gallate, epigallocatechin, epigallocatechin gallate, gallocatechin, kaempferol, kaempferol-3-glucuronide, quercetin, quercetin-3-glucuronide, quercetin-3-sulfate, and rutin. Carotenoid analysis included *β*-carotene, lycopene, astaxanthin, lutein, and zeaxanthin. The organic acids (acetic acid, citric acid, fumaric acid, lactic acid, malic acid, oxalic acid, propionic acid, succinic acid, and tartaric acids) were analyzed in peel samples. The water-soluble vitamins included thiamine, riboflavin, niacin, pantothenic acid, pyridoxine, biotin, folic acid, cobalamin, and ascorbic acid.

UHPLC-MS/MS analyses were performed using a Thermo Scientific Ultimate 3000 UHPLC system coupled to a Thermo Scientific TSQ Fortis triple quadrupole mass spectrometer (Thermo Fisher Scientific Inc., Waltham, MA, USA). Chromatographic separation was performed using a Hypersil GOLD RP-C18 column (1.9 µm, 50 × 2.1 mm). The column temperature was maintained at 40 °C, and the injection volume was 10 µL. The capillary temperature of the mass spectrometer was set to 270 °C. The mobile phases consisted of (A) water/methanol (95:5, *v*/*v*) and (B) water/methanol (5:95, *v*/*v*), both containing 4 mM ammonium formate and formic acid. The gradient elution program was as follows: 0–4 min, 100% A; 4–4.5 min, 50% A; 4.5–6 min, 0% A; and 6–7 min, 100% A. The flow rate was maintained at 0.3 mL min-1. The electrospray ionization (ESI) source was operated at a vaporizer temperature of 50 °C, a sheath gas pressure of 50 Arb, an auxiliary gas pressure of 20 Arb, and a spray voltage of ±3500 V. Data collection and evaluation were performed using Xcalibur software (version 4.7). Metabolite identification was performed by comparing the retention times and mass spectra of the analytes with those of analytical-grade reference standards. Quality control was performed throughout the UHPLC-MS/MS analytical sequence. A pooled quality-control sample was injected at regular intervals during the analysis to monitor instrumental stability, including retention-time consistency and signal response. Peak-area relative standard deviation values obtained from the quality-control injections were evaluated according to the predefined quality-control acceptance criteria of the analytical laboratory. All samples representing the six developmental stages were analyzed within a single analytical batch/sequence under the same conditions.

### 2.7. Aroma and Volatile Compound Analysis

Aroma and volatile compounds present in the lyophilized methanolic orange peel extracts were analyzed using gas chromatography-mass spectrometry (GC-MS). For sample preparation, 0.5 g of the lyophilized methanolic extract was mixed with 9.5 mL of acetone. The resulting mixture was vortexed for 5 min and centrifuged at 4000 rpm for 15 min at 4 °C. The supernatant was subsequently diluted at a ratio of 1:2 and transferred to 2 mL vials for GC-MS analysis. GC-MS analyses were performed using a Thermo Scientific Trace 1300 gas chromatograph coupled to a Thermo Scientific ISQ7000 single quadrupole mass spectrometer (Thermo Fisher Scientific Inc., Waltham, MA, USA). Chromatographic separation was achieved using a TraceGOLD^TM^ TG-624 SilMS capillary column (30 m × 0.32 mm; 1.8 µm film thickness). The injection temperature was set to 250 °C, and samples were injected at a 1:5 split ratio with an injection volume of 5 μL. Helium was used as the carrier gas at a flow rate of 1.5 mL min^−1^. The oven temperature was initially set to 30 °C and held for 3 min, then increased to 230 °C at a rate of 5 °C min^−1^ and held for 5 min. The detector temperature was set to 280 °C, and the same operating conditions were applied to all samples. Volatile compounds were identified by comparing their mass spectra with reference spectra in the National Institute of Standards and Technology (NIST) mass spectral libraries NIST27 and NIST147. Molecular and characteristic ions were determined for each identified compound. Quantitative evaluation of the identified compounds was performed by comparing the analytical responses of the samples with those obtained from analytical-grade representative standards at known concentrations.

### 2.8. Mineral Composition Analysis

The mineral composition of lyophilized methanolic extracts was determined using inductively coupled plasma optical emission spectrometry (ICP-OES). For sample preparation, 0.5 g of the lyophilized methanolic extract was subjected to microwave-assisted acid digestion with 8 mL of nitric acid and 2 mL of hydrogen peroxide using an ETHOS ONE microwave sample preparation system (Milestone, Sorisole, Bergamo, Italy). The digestion procedure was performed according to EPA 3015. Following digestion, the volume was adjusted to 20 mL with distilled water. Element concentrations were determined using an Optima 5300 DV ICP-OES spectrometer (PerkinElmer, Waltham, MA, USA) according to EPA 6010 [18]. The analyzed elements and wavelengths were 317.9 nm for calcium (Ca), 327.4 nm for copper (Cu), 238.2 nm for iron (Fe), 766.5 nm for potassium (K), 285.2 nm for magnesium (Mg), 257.6 nm for manganese (Mn), 589.6 nm for sodium (Na), 213.6 nm for phosphorus (P), 196.0 nm for selenium (Se), 251.6 nm for silicon (Si) and 206.2 nm for zinc (Zn) and the results were given as mg g^−1^ dry weight (mg g^−1^ DW).

### 2.9. Measurement of Antibacterial Activity

The antibacterial activity of lyophilized orange peel methanolic extracts was evaluated against Gram-negative bacterium *Escherichia coli* (ATCC 25922) and the Gram-positive bacteria *Enterococcus faecalis* (ATCC 29212), *Staphylococcus aureus* (ATCC 29213) and *Bacillus cereus* (ATCC 11778) using the agar well diffusion method [19,20]. Bacterial cultures were adjusted to a 0.5 McFarland standard, corresponding to approximately 1.5 × 10^8^ CFU mL^−1^. A 100 µL aliquot of each bacterial suspension was spread onto sterile Petri plates containing soft Luria–Bertani agar (LBA; 0.5% agar) as the upper layer and allowed to dry. After solidification, wells with a diameter of 6 mm were aseptically prepared using a sterile cork borer. Lyophilized methanolic extracts from each developmental stage were prepared at concentrations of 500, 250, and 125 mg mL^−1^ in pure dimethyl sulfoxide (DMSO), and 50 µL of each extract solution was added to the wells. The plates were incubated at 37 °C for 24 h. Antibacterial activity was evaluated by measuring the diameter of the clear inhibition zones around the wells and expressed in millimeters. Gentamicin (10 µg) for Gram-negative bacteria and vancomycin (30 µg) for Gram-positive bacteria were used as positive controls, while pure DMSO was used as the negative control. Pure DMSO was used as the solvent control to assess any potential antibacterial effect of the solvent itself. All experiments were performed in three independent experiments, each with three technical replicates, and the results were expressed as mean ± standard deviation.

The minimum inhibitory concentration (MIC) and minimum bactericidal concentration (MBC) of the lyophilized methanolic extracts obtained from each developmental stage were determined using the broth dilution method. Bacterial suspensions were prepared and adjusted to a 0.5 McFarland standard, corresponding to approximately 1.5 × 10^8^ CFU mL^−1^. Each lyophilized extract was serially diluted two-fold in pure DMSO and added to 1 mL of LB broth to obtain final test concentrations ranging from 500 to 0.03 mg mL^−1^. Pure DMSO solution was used as the negative control. The tubes were incubated at 37 °C for 24 h. The MIC was defined as the lowest extract concentration at which no visible bacterial growth was observed. For MBC determination, 5 µL aliquots from tubes showing no visible growth were inoculated onto LB agar plates and incubated at 37 °C for 24 h. The MBC was defined as the lowest extract concentration that resulted in no visible bacterial growth on the agar plates. All experiments were performed in three independent experiments, each with three technical replicates.

### 2.10. Statistical Analysis

All analytical results were expressed as mean ± standard deviation (SD) of three independent replicates. Statistical analyses were performed using SPSS Statistics version 27 (IBM, Armonk, NY, USA). One-way analysis of variance (ANOVA) was performed to determine statistically significant differences among developmental stages, followed by Duncan’s multiple range test at a significance level of *p* ≤ 0.05. For heatmap visualization, metabolite abundances were standardized across developmental stages by row-wise *Z*-score transformation, allowing relative changes in each metabolite to be compared across stages. Heatmaps were generated using TBtools-II (v2.542). The identified metabolites were subsequently mapped to the corresponding reference metabolic pathways in the KEGG database (map00941, map00940, map00906, map00902, map00760, map00740, map00730, map00630, map00053 and map00020). Principal component analysis (PCA) was performed to assess multivariate patterns in the metabolite dataset, which included 38 variables comprising 32 UHPLC-MS/MS-determined metabolites and 6 GC-MS-determined volatile compounds. The 18 observations, corresponding to three biological replicates from each developmental stage, were analyzed using the correlation matrix. Pearson correlation analysis was performed to assess relationships among pomological characteristics, biochemical parameters, selected metabolites, and antibacterial activity. PCA and Pearson correlation analysis were calculated using OriginPro 2026b (OriginLab Corporation, Northampton, MA, USA).

## 3. Results and Discussion

### 3.1. Pomological Characteristics

**Figure 2:**
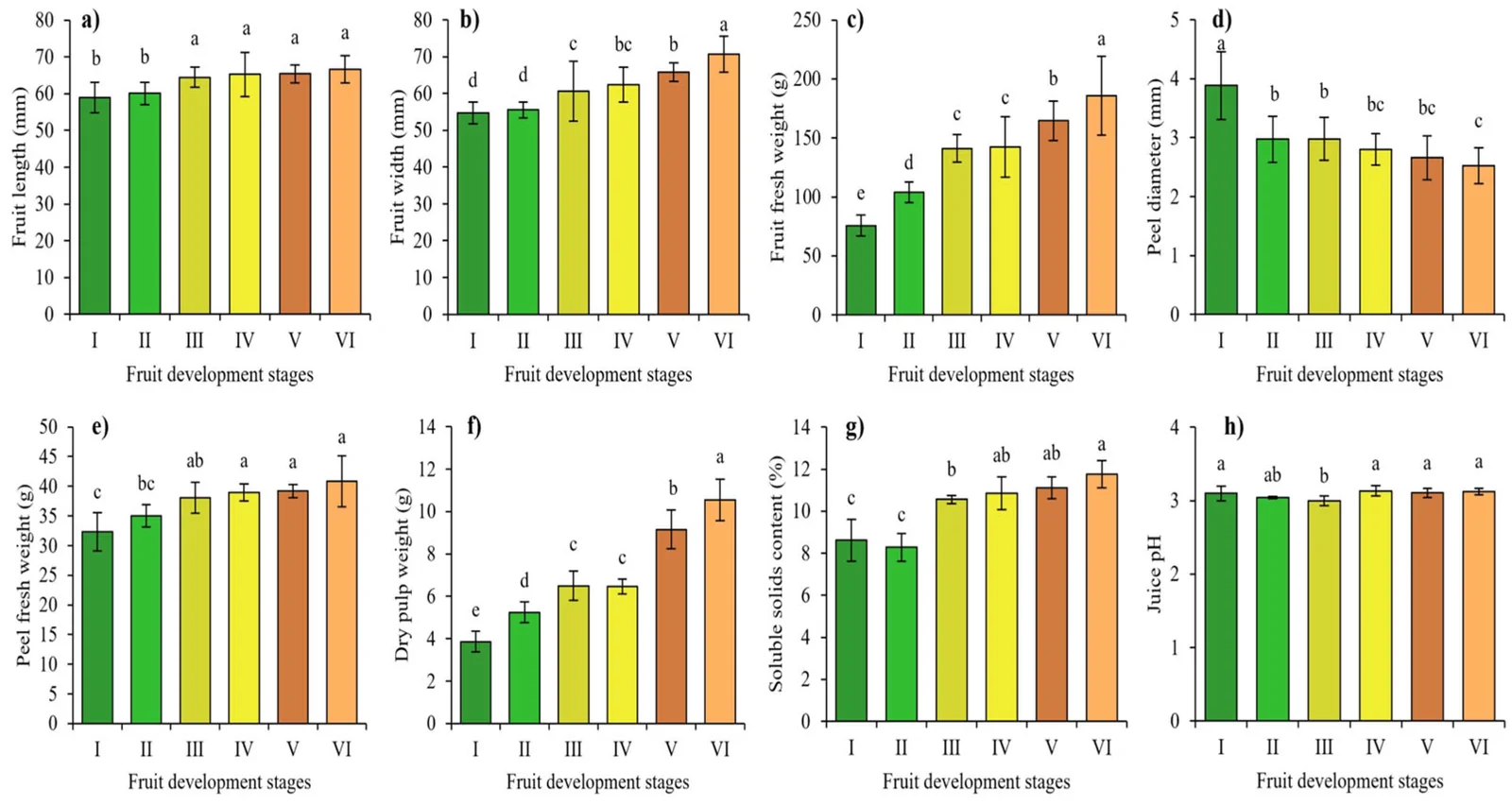
Changes in pomological variables of Valencia across six sampling dates corresponding to different development stages of fruits. Fruit length (**a**), fruit width (**b**), fruit fresh weight (**c**), peel thickness (**d**), peel fresh weight (**e**), dry pulp weight (**f**), soluble solids content (**g**) and juice pH (**h**). Data are presented as mean ± standard deviation. Different lowercase letters indicate significant differences among developmental stages according to Duncan’s multiple range test (*p* ≤ 0.05).

The pomological variables examined throughout fruit development revealed that fruit growth and ripening in Valencia oranges involve a series of successive processes (Figure 2). Fruit length, fruit width, fruit fresh weight, peel fresh weight, dry pulp weight and soluble solids content generally increased across the developmental stages, whereas peel thickness decreased relative to the initial sampling. In contrast, juice pH showed comparatively limited variation throughout development. In general, these changes indicate that cell division, growth and tissue development during the fruit enlargement phase contributed to increases in fruit size and weight, whereas during ripening fruit quality characteristics, such as changes and accumulation of bioactive compound profiles, were more important [21,22].

Fruit length and width increased at different rates during development. Fruit length was 58.94 ± 4.09 mm in Stage I, reaching to 64.45 ± 2.71 mm in Stage III and did not change significantly at subsequent stages (Figure 2a). In contrast, fruit width increased from 54.73 ± 3.0 mm in Stage I to 70.70 ± 4.91 mm in Stage VI, representing an increase of approximately 29.2% during development (Figure 2b). Therefore, the increase in width was more pronounced than the increase in length at later stages of development. A similar developmental trend has also been reported in studies examining fruit diameter and height throughout development in different orange varieties [23]. However, fruit shape and growth rates may vary depending on the variety, environmental conditions, and cultural practices [21].

Fruit fresh weight increased significantly during development, rising from 75.80 ± 8.95 g in Stage I to 185.67 ± 33.49 g in Stage VI (Figure 2c). Thus, fruit fresh weight increased by approximately 144.9% from the first to sixth sampling times. The similar values observed in Stages III and IV indicate that the increase in fruit mass was limited between these stages, and that a significant increase in mass occurred again at the subsequent sampling times. Ferrer et al. [24] also reported that fruit weight generally increased throughout development in different orange varieties. The trend observed in the current study is consistent with the reported developmental model.

Peel thickness steadily decreased throughout fruit development, from 3.89 ± 0.58 mm in Stage I to 2.66 ± 0.37 mm in Stage VI, resulting in a total decrease of approximately 31.6% (Figure 2d). In contrast, peel fresh weight increased from 32.33 ± 3.22 g in Stage I to 38.06 ± 2.57 g in Stage III, and 40.85 ± 4.31 g in Stage VI (Figure 2e). The statistically similar peel fresh weight values observed at later stages (Stages IV–VI) indicate that mass gain in the peel tissue remains limited during these sampling times. The increase in peel fresh weight despite the decrease in peel thickness indicates that changes in peel dimensions were not limited to simple thickening during these stages. Similar developmental behavior has been described anatomically in Valencia orange, where rapid fruit growth was associated primarily with cell enlargement, while the peel became thinner as pulp growth continued [22]. Furthermore, peel and pulp development in citrus fruits are not entirely simultaneous and these tissues may follow partially independent developmental processes [21]. The consistent increase in fruit fresh weight throughout fruit development further suggests that total weight gain may be more closely associated with the development of internal tissues rather than peel tissue.

The potential relationship between increased fruit fresh weight and development of internal tissues is further supported by the significant increase in dry pulp weight across the sampling dates. Dry pulp weight increased throughout development, reaching 3.86 ± 0.49 g in Stage I, 5.24 ± 0.48 g in Stage II, 6.50 ± 0.70 g in Stage III, 6.46 ± 0.35 g in Stage IV, 9.15 ± 0.91 g in Stage V, and finally 10.54 ± 0.97 g in Stage VI (Figure 2f). Thus, dry pulp weight increased by approximately 173.1% between Stages I and VI. The significant increases observed throughout the development indicate that dry matter accumulation is a continuous process during fruit development. The increase in dry pulp weight, together with the overall increase in fruit fresh weight, suggests that the growth of internal tissues and accumulation of soluble solids play a significant role at later stages of fruit development [25].

Soluble solids content did not change significantly from Stage I (8.62 ± 1.00%) to Stage II (8.28 ± 0.66%) (Figure 2g). From Stage III onward, soluble solids content increased steadily, reaching its highest value of 11.76 ± 0.64% in Stage VI. It has been reported that the soluble solids content of ripe oranges could range from 8–13% depending on the variety, and that Valencia also falls within this range [21]. Soluble solids content values in two different *C. sinensis* varieties and in Valencia fluctuate during the early developmental stages 7.8 °Brix before reaching to approximately 12 °Brix at later stages [23,24]. The value obtained in Stage IV of the current study is consistent with the previous reports.

In contrast, fruit juice pH showed very limited variation throughout the study period. The juice pH decreased from 3.10 ± 0.10 in Stage I to 3.00 ± 0.06 in Stage III, after which it increased to 3.13 ± 0.07 in Stage IV. No significant difference was observed between Stage IV and Stage VI (Figure 2h). These results indicate that Valencia orange juice remained acidic throughout fruit development. Organic acid metabolism and, consequently, fruit juice during fruit development and ripening may vary depending on citrus species and varieties [21]. The juice pH of approximately 3.47 was reported for Valencia oranges [26] and lower pH values than the reported values suggest that fruit pH is affected by growing site and other environmental factors. Overall, the observed changes indicate that fruit development in Valencia orange involved increases in fruit size, weight and dry matter accumulation.

### 3.2. Total Phenolics, Flavonoid Content and Antioxidant Activity

**Figure 3:**
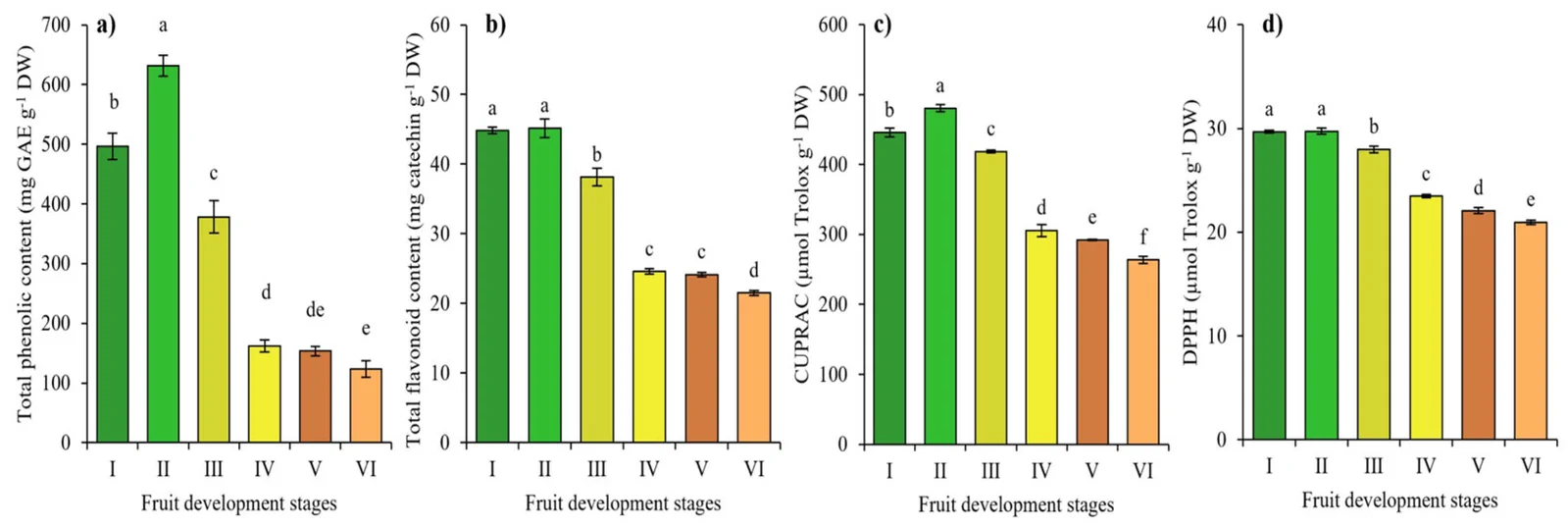
Changes in total phenolic content (**a**), total flavonoid content (**b**), and CUPRAC (**c**) and DPPH (**d**) antioxidant activities at different stages. Data are presented as mean ± standard deviation. Different lowercase letters indicate significant differences among developmental stages according to Duncan’s multiple range test (*p* ≤ 0.05).

Citrus peels are rich in secondary compounds, particularly phenolic acids and flavonoids. Previous studies have shown that citrus peels contain higher levels of phenolic compounds and flavonoids than other fruit tissues [27,28]. These compounds are also considered important contributors to the antioxidant properties of citrus fruits [29]. In the present study, total phenolics, flavonoid content and antioxidant activity varied significantly across the study period in orange peel (Figure 3).

Total phenolics content varied significantly among the developmental stages. The highest total phenolics content (631.47 ± 17.5 mg GAE g^−1^ DW) was observed in Stage II, increasing significantly from 496.36 ± 22.03 mg GAE g^−1^ DW in Stage I (Figure 3a). After Stage II, total phenolics content steadily decreased significantly at Stage III, Stage IV and Stage VI, decreasing to 123.73 ± 13.76 mg GAE g^−1^ DW at Stage VI. Thus, total phenolics content in Stage VI decreased by approximately 80.4% decrease was recorded from Stage II to Stage VI during the study period. The significant decrease is consistent with previous reports where phenolic compound levels decreased as the development of orange fruits progressed. Hou et al. [11] reported that total phenolic content in navel orange (*C. sinensis* cv. Newhall) pulp extracts decreased significantly from the young fruit stage to commercial maturity. Similarly, Wang et al. [7] showed that total phenolics content in brocade orange (*C. sinensis* L. Osbeck) peel was higher during the early and semi-ripe stages and was lower at commercial ripening. Total phenolics content was generally higher in peel than in pulp and phenolic substances decreased markedly during fruit ripening, although occurrence of increases during post-harvest cold storage have also been reported [30,31]. Overall, phenolics composition is highly dependent on fruit type, development stage and growing conditions. Furthermore, oxidation of polyphenols by polyphenol oxidase may have contributed to the decrease in total phenolics content during fruit ripening and sample preparation [32].

Flavonoids are an important group of polyphenolic compounds and contribute to the antioxidant properties of citrus fruits [8]. Total flavonoid content did not change between Stage I (44.83 ± 0.46 mg catechin g^−1^ DW) and Stage II (45.17 ± 1.31 mg catechin g^−1^ DW) but dropped significantly at Stage III (38.13 ± 1.26 mg catechin g^−1^ DW) and Stage IV (24.57 ± 0.40 mg catechin g^−1^ DW) compared with Stage II (Figure 3b). Total flavonoid content did not differ significantly between Stage IV and Stage V but significantly dropped at Stage VI (21.56 ± 0.36 mg catechin g^−1^ DW). Thus, compared to Stage II, where total flavonoid content was highest, its amount decreased by approximately 52.4% at Stage VI, which was also observed in different citrus species and varieties; a decrease in total flavonoid content was reported as development progressed [21,33]. Hou et al. [11] and Wang et al. [7] showed that total phenolics and flavonoid content in orange peels were higher, particularly at early and semi-mature stages. However, there are studies reporting lower total flavonoid content in peel extracts [8,34]. These differences should take into consideration variety, harvest times, extraction solvent, solvent composition, extraction conditions, and the colorimetric methods used. Indeed, it has been reported that solvent type, composition, and extraction conditions affect the total yield of phenolic compounds [35] and that total flavonoid content in orange peel extracts varied significantly depending on the solvent system used [4]. Therefore, in the present study, total flavonoid content was evaluated across different developmental stages rather than comparing total flavonoid content values among different studies.

The antioxidant activity of orange peel extracts at different stages was evaluated using the CUPRAC and DPPH assays. CUPRAC activity exhibited a trend similar to that of total phenolics content. CUPRAC activity increased from 445.58 ± 6.05 μmol Trolox g^−1^ DW in Stage I to the highest value of 480.41 ± 5.07 μmol Trolox g^−1^ DW in Stage II (Figure 3c). In subsequent sampling times, CUPRAC activity decreased significantly at each stage to 418.48 ± 2.12, 305.61 ± 8.66, 292.25 ± 0.93 and 263.45 ± 4.80 μmol Trolox g^−1^ DW in Stages III, IV, V and VI, respectively. Accordingly, CUPRAC activity decreased by approximately 45.2% from its highest value at Stage II to its lowest value at Stage VI. A similar trend was reported, where antioxidant activities measured by ABTS, FRAP and DPPH assays decreased as development progressed in navel orange [11].

DPPH radical scavenging activity also varied significantly and generally followed a trend similar to that of CUPRAC activity (Figure 3d). Although DPPH activity increased from 26.69 ± 0.12 μmol Trolox g^−1^ DW in Stage I to 29.74 ± 0.28 μmol Trolox g^−1^ DW in Stage II, the increase was not significant. DPPH activity at subsequent sampling times showed a significant decrease from 27.98 ± 0.31 μmol Trolox g^−1^ DW in Stage III to 20.95 ± 0.20 μmol Trolox g^−1^ DW in Stage VI. Thus, DPPH activity decreased by approximately 29.6% from Stage II to Stage VI. This result is consistent with Hou et al. [11], who reported a decrease in DPPH radical scavenging activity during fruit development and ripening in navel orange.

The observed results represent an overall response obtained by methanolic extracts; however, these measurements did not reveal how individual phenolic acids, flavonoids and carotenoids contributed to the observed patterns in Figure 3. Therefore, profiles of individual compounds were further evaluated using targeted chromatographic analysis.

### 3.3. Profiles of Phenolic Acids, Flavonoids, and Carotenoids in Orange Peel Extracts

Citrus fruits possess a wide variety of carotenoids, which play significant roles in the formation of the characteristic color of fruit flesh and peel [36,37,38]. In the present study, carotenoids showed the largest total increase throughout development compared to flavonoids and phenolic acids. Total carotenoid concentration was 4569.5 mg kg^−1^ in Stage I and reached its highest level of 10,277.8 mg kg^−1^ in Stage VI (Table 3). *β*-Carotene was the major carotene, with its concentration rising from 3748.2 mg kg^−1^ in Stage I to 8275.9 mg kg^−1^ in Stage III and reaching its amount of 8596.4 mg kg^−1^ in Stage VI. Although lycopene was also present in lower concentrations, it showed a steady significant increase throughout the study period, rising from 127.3 mg kg^−1^ in Stage I to 210.2 mg kg^−1^ in Stage VI. Within the xanthophyll group, lutein and zeaxanthin exhibited a similar pattern of accumulation within peel samples, where lutein and zeaxanthin concentrations increased from Stage I to Stage IV, then decreased at Stage VI. Astaxanthin was not detected in peel samples. These results demonstrate that carotenoids in orange peel exhibit compound-specific accumulation patterns distinct from those of flavonoids and phenolic acids throughout development.

The marked increase in carotenoid concentrations from Stage III onwards coincided with the visually observed progression of peel coloration (Figure 1), particularly the accumulation of *β*-carotene. Rodrigo et al. [39] reported that significant changes in carotenoid content and composition occur with the onset of coloration in the flavedo of orange fruit, with carotenoid accumulation increasing rapidly after the onset of coloration. In the present study, the marked increase in total carotenoid concentration from Stage III onwards, with *β*-carotene as the major contributor, was consistent with the progression of visible peel coloration. Lutein and zeaxanthin reached high levels in Stage IV and then followed different patterns in subsequent stages, indicating that carotenoids do not accumulate uniformly and individual pigments have different accumulation patterns. Citrus peel pigmentation is associated with coordinated changes in chlorophyll degradation and carotenoid accumulation, with carotenoids forming the main pigment group involved in the development of the characteristic color of ripe fruit [36,40]. Carotenoid metabolism is also linked to ABA biosynthesis because ABA is derived from carotenoid precursors, providing a biochemical connection between pigment metabolism and ripening regulation. In Valencia orange, carotenoid accumulation and peel coloration during ripening are associated with ABA accumulation and exogenous ABA treatment also promoted both peel coloration and carotenoid accumulation [41].

Phenolic acids, flavonoids and carotenoids are important compounds commonly found in fruits and vegetables, valuable sources of bioactive compounds due to their diverse phytochemical composition [7,21,42]. Numerous phenolic acids and flavonoids have been identified in citrus peels using UHPLC-MS/MS, with narirutin, naringin, hesperidin and other flavonoid derivatives reported to be important components [43,44,45]. Shehata et al. [8] identified various phenolic and flavonoid compounds in orange peel extracts and showed that their profiles varied depending on the extraction conditions.

UHPLC-MS/MS analyses quantitatively identified 21 of the 34 compounds, consisting of 4 carotenoids, 11 flavonoids and 6 phenolic acids (Table 3). The total concentration of these compounds increased from 46,955.6 mg kg^−1^ in Stage I to 82,776.8 mg kg^−1^ in Stage V, then decreased to 66,555.3 mg kg^−1^ in Stage VI. Flavonoids represented the compound class with the highest total concentration among the quantitatively determined compounds. Total flavonoid concentration increased from 34,343.2 mg kg^−1^ in Stage I to the highest value of 60,169.0 mg kg^−1^ in Stage V then decreased to 44,038.6 mg kg^−1^ in Stage VI. Total flavonoid concentration did not show an increase when measured with a spectrophotometer (Figure 3b), but it was found to be increased when a more specific analytical procedure was used. The difference between these trends does not represent a direct contradiction since the two analytical approaches measure different aspects of the flavonoid pool. The aluminum chloride colorimetric assay provides a global estimate expressed as catechin equivalents and may be influenced by the different response characteristics of individual flavonoids [46]. In contrast, the UHPLC-MS/MS values represent the sum of the concentrations of the 11 flavonoids that were quantitatively determined using specific analytical standards. Therefore, the values obtained by the two methods are not directly interchangeable and are not expected to show identical developmental trends.

Among the individual flavonoids, hesperidin was the most abundant one throughout all stages and showed significant changes during fruit development. Hesperidin concentration changed significantly between sampling times and reached its highest concentration of 47,161.0 mg kg^−1^ in Stage III, up from 27,728.5 mg kg^−1^ in Stage I and later decreased to 32,050.5 mg kg^−1^ in Stage VI (Table 3). Naringin content showed a similar accumulation pattern to hesperidin content. Naringin content reached its highest value at Stage III (6428.5 mg kg^−1^) and at subsequent sampling times it had lower concentration in orange peel. In contrast to hesperidin and naringin accumulation patterns, narirutin concentration increased significantly from 1257.9 mg kg^−1^ in Stage I to 3674.0 mg kg^−1^ in Stage V and then decreased significantly to 3157.2 mg kg^−1^ in Stage VI. These differences among flavonoids suggest that flavonoid biosynthesis is not a constant process; rather, the synthesis, conversion and accumulation of individual flavonoids are regulated independently [47,48]. Similarly, flavonoids exhibited different accumulation profiles throughout development in orange peels, with flavanones being particularly important components [7]. Furthermore, hesperidin has been reported as one of the major flavonoids occurring at high concentrations in orange peels and other *Citrus* species [7,49]. Some reports suggested that hesperidin possesses various pharmacological activities [50], perhaps most promising being its potential to provide protection against COVID-19 [51]. In the present study, hesperidin was identified as a key component of the flavonoid pool throughout fruit development.

Besides hesperidin, naringin, and narirutin, other flavonoid compounds also showed different accumulation patterns. Apigenin-7-glucoside, catechin, kaempferol-3-glucuronide, quercetin and rutin accumulation were similar to the above individual flavonoids, while apigenin and kaempferol showed a steady increase and epicatechin showed a steady decrease throughout fruit development. For example, apigenin concentration increased from 226.8 mg kg^−1^ in Stage I to 422.1 mg kg^−1^ in Stage VI, while epicatechin content decreased throughout the developmental period, from 284.7 mg kg^−1^ in Stage I to 247.7 mg kg^−1^ in Stage VI. Eight flavonoid compounds (cyanidin, delphinidin, epicatechin gallate, epigallocatechin, epigallocatechin gallate, gallocatechin, quercetin 3 glucuronide, quercetin 3 sulfate) were not detected in orange peels throughout fruit development. These differences suggest that individual flavonoids in orange peel do not exhibit a common trend of change throughout fruit development and that the developmental accumulation patterns of the compounds are compound-specific. Phenolics may occur as free, esterified, glycosylated, and insoluble-bound forms, and these fractions can undergo different changes during fruit growth and ripening [7,52]. In addition, polyphenol oxidase is present in *C. sinensis* and can catalyze the oxidation of phenolic substrates to quinones, which may subsequently undergo polymerization and reduce the pool of extractable phenolic compounds [53]. Therefore, the increase in selected phenolic acids observed in the present study does not necessarily imply a corresponding increase in the overall phenolic pool as measured by the Folin–Ciocalteu assay.

The total concentration of phenolic acids increased steadily and reached the highest value at Stage V (12,781.1 mg kg^−1^), then decreased to 12,238.9 mg kg^−1^ in Stage VI (Table 3). Among the phenolic acids, ferulic, caffeic, vanillic, and *p*-coumaric acids were the most abundant compounds. Ferulic acid concentration increased from 2765.3 mg kg^−1^ in Stage I to 5581.2 mg kg^−1^ in Stage V. Caffeic acid, on the other hand, increased steadily throughout the developmental period, rising from 2524.1 mg kg^−1^ in Stage I to 3391.8 mg kg^−1^ in Stage VI. *p*-Coumaric acid also reached its highest level of 1545.4 mg kg^−1^ in Stage V. Vanillic acid content also increased from Stage I to Stage IV and decreased at later stages. In contrast, syringic acid concentration was highest at Stage I (363.2 mg kg^−1^) and decreased to 257.8 mg kg^−1^ in Stage VI. Ellagic acid, unlike other phenolic acids, increased continuously, rising from 278.5 mg kg^−1^ in Stage I to 315.5 mg kg^−1^ in Stage VI. Rosmarinic, caftaric, chlorogenic and gallic acids were not detected in peel samples from different sampling times.

Hou et al. [11] reported a maturation-related decrease in most phenolic acid components in the pulp of navel orange fruit throughout development, whereas different trends were observed for ferulic and caffeic acids. In the same study, ferulic acid was the most abundant compound among the phenolic acids, as in the present study. Wang et al. [7] also showed that the changes in phenolic acids, such as ferulic and *p*-coumaric acids in orange peels, varied depending on the peel tissue and extraction method. Ferulic acid is considered a versatile bioactive compound that provides health benefits and may act as a protective factor against diseases associated with reactive oxygen species, such as cancer, cardiovascular diseases, and diabetes [11,54]. However, since the developmental profiles of phenolic acids can vary depending on fruit tissue and compound, direct comparisons of concentrations and developmental trends among different studies should be made with caution.

In addition, since the peel is directly exposed to the environment, tissue may be more responsive to environmental factors, which may affect the accumulation of phenolic compounds. This may be one reason why plants, including fruits, generally store higher concentrations of phenolic compounds in protective tissues (e.g., peel, seed coat, outer membrane) than in nutritive tissues (e.g., flesh, embryo, endosperm), and why young or immature fruits have much higher resistance to biotic and abiotic stresses than mature fruits [54].

### 3.4. Organic Acids, Vitamins and Aroma-Volatile Compounds

The presence of 9 organic acids was investigated and six were present while three (acetic, lactic and propionic acids) were absent in orange peels. The presence of organic acids in orange peel showed significant variation throughout the sampling period (Table 4). Citric and malic acids were the predominant organic acids, while citric acid had the highest concentration throughout all developmental stages, increasing from 37,284.7 mg kg^−1^ in Stage I to 55,266.4 mg kg^−1^ in Stage III and decreasing to 41,227.5 mg kg^−1^ in Stage VI. Malic acid increased from 8908.0 mg kg^−1^ in Stage I to 17,119.1 mg kg^−1^ in Stage IV, before decreasing to 13,089.5 and 13,080.4 mg kg^−1^ in Stages V and VI, respectively. Oxalic, tartaric, fumaric and succinic acids also showed similar accumulation patterns to citric and malic acids; their concentrations increased significantly at early stages and decreased significantly at later stages of fruit development. Succinic and fumaric acids were detected at the lowest concentrations. Total organic acid concentration increased by 55.8% from Stage I to Stage III, reaching 74,468.1 mg kg^−1^, and then decreased in subsequent stages, dropping to 58,690.5 mg kg^−1^ in Stage VI. These results revealed that citric and malic acids are key components of the total organic acid profile present in orange peel.

Feng et al. [55] reported that citric and malic acid content in orange pulp reached their maximum at 80 DAF and then decreased to 220 and 120 DAF, respectively. However, malic acid content increased from 120 to155 DAF. They also showed that organic acid accumulation is regulated by the coordination of synthesis, degradation, and vacuolar/mitochondrial transport processes. Liew et al. [4] reported that citric and malic acids were present in high amounts in orange peel extracts and that citrus-derived organic acids can be used in food, beverage, pharmaceutical and cosmetic applications. Given that the global supply of organic acids, particularly citric acid, was 2.39 million tons in 2020 and is projected to reach 2.91 million tons in 2026 [56], obtaining organic acids from citrus industrial waste could represent a potential alternative source of natural organic acids. However, the fact that total organic acid concentrations remained high in mature peel despite the decrease observed during the later developmental stages in the present study indicates that the peel retains its potential as an important source material for organic acids at the harvesting stage.

Water-soluble vitamins, such as thiamin (B_1_), riboflavin (B_2_), niacin (B_3_), pyridoxine (B_6_) and ascorbic acid (C) were detected throughout the study period, whereas pantothenic acid (B_5_), biotin (B_7_), folic acid (B_9_) and cobalamin (B_12_) levels were below the detection limit at all developmental stages (Table 5). The undetected vitamins should not necessarily be interpreted as their complete absence from orange peel, as B-group vitamins, including folate and pantothenic acid, have been reported among the micronutrients present in citrus fruits and orange-derived by-products [57,58]. In particular, vitamin B_12_ is not considered a normal constituent of unfortified plant foods and is primarily synthesized by microorganisms [59]. Ascorbic acid was the main vitamin and its concentration increased significantly from Stage I to Stage V, an increase of 39.8% from 1078.5 mg kg^−1^ (Stage I) to 1508.3 mg kg^−1^ (Stage V), with a slight but not significant decrease to 1487.6 mg kg^−1^ (Stage VI). Thiamin concentration was 9.73 mg kg^−1^ in Stage I, and gradually increased, reaching 14.42 mg kg^−1^ in Stage VI. Similarly, niacin concentration increased from 6.41 mg kg^−1^ (Stage I) to 11.92 mg kg^−1^ (Stage VI). Although riboflavin and pyridoxine concentrations showed slight fluctuations, both reached their highest levels in Stage V. Alós et al. [60] reported that ascorbic acid content of orange fruit reaches higher levels during the discoloration period of peel, whereas ascorbic acid content of pulp tissues remains low and shows limited fluctuations throughout development. Metabolic profiles of citrus peel and pulp are not entirely synchronous and some metabolites, such as ascorbic acid and carotenoids, may accumulate at higher levels in peel than the other metabolites [21].

*Citrus* fruits have a complex aroma and volatile compound composition, comprising a heterogeneous mixture of volatile compounds, most of which belong to the terpene family. During fruit ripening, significant qualitative and quantitative changes occur in the aromatic profile, which becomes abundant and diverse during the early stages of fruit development and color change [21,33]. The profile of aroma-volatile compounds in orange peel showed significant changes in both concentration and composition throughout fruit development (Table 6). Citrus peels generally contain more than 70% limonene [61], and this compound gives a distinct citrus aroma; limonene could also be used as an insect repellent [62]. As with the previous studies, limonene was the main volatile compound detected at all stages, increasing significantly from 7.71 mg kg^−1^ in Stage I to 13.43 mg kg^−1^ in Stage II and reaching its peak of 27.22 mg kg^−1^ in Stage III. Thereafter, limonene concentration decreased significantly at subsequent stages, dropping to 14.71 mg kg^−1^ in Stage VI. Because the present analysis was conducted on a methanolic extract rather than isolated essential oil, the absolute limonene concentration should not be directly compared with the relative composition (%) reported for citrus peel essential oils.

Citral was also present in lower quantities and reached its maximum concentration of 0.33 mg kg^−1^ in Stage III and decreased to 0.16 mg kg^−1^ in Stage VI. *α*-Pinene and geraniol were only detected in Stage III, at concentrations of 0.11 and 0.14 mg kg^−1^, respectively, whereas linalool was detected in Stages III and IV, and myrcene was detected in Stages III-V. These results demonstrate that the aroma-volatile profile changed not only quantitatively but also compositionally. Similarly, Xie et al. [13] examined approximately 60 volatile compounds in orange peels at different fruit developmental stages and reported that most volatile components increased during the early stages of development, decreased during the middle stages, and some compounds also increased again at maturity. In the same study, *d*-limonene was identified as the main component throughout the developmental stages. Ferrer et al. [24] reported that limonene was the dominant component in the peel essential oils of two sweet orange varieties and decreased after reaching its highest concentration at Stage III. In the present study, limonene and citral reached their maximum levels at Stage III, suggesting that the middle stages of development represent an important transition point in the volatile metabolite profile of orange peel. In addition, these compounds have important applications in the aroma, fragrance, aromatherapy and agrochemical industries.

**Figure 4:**
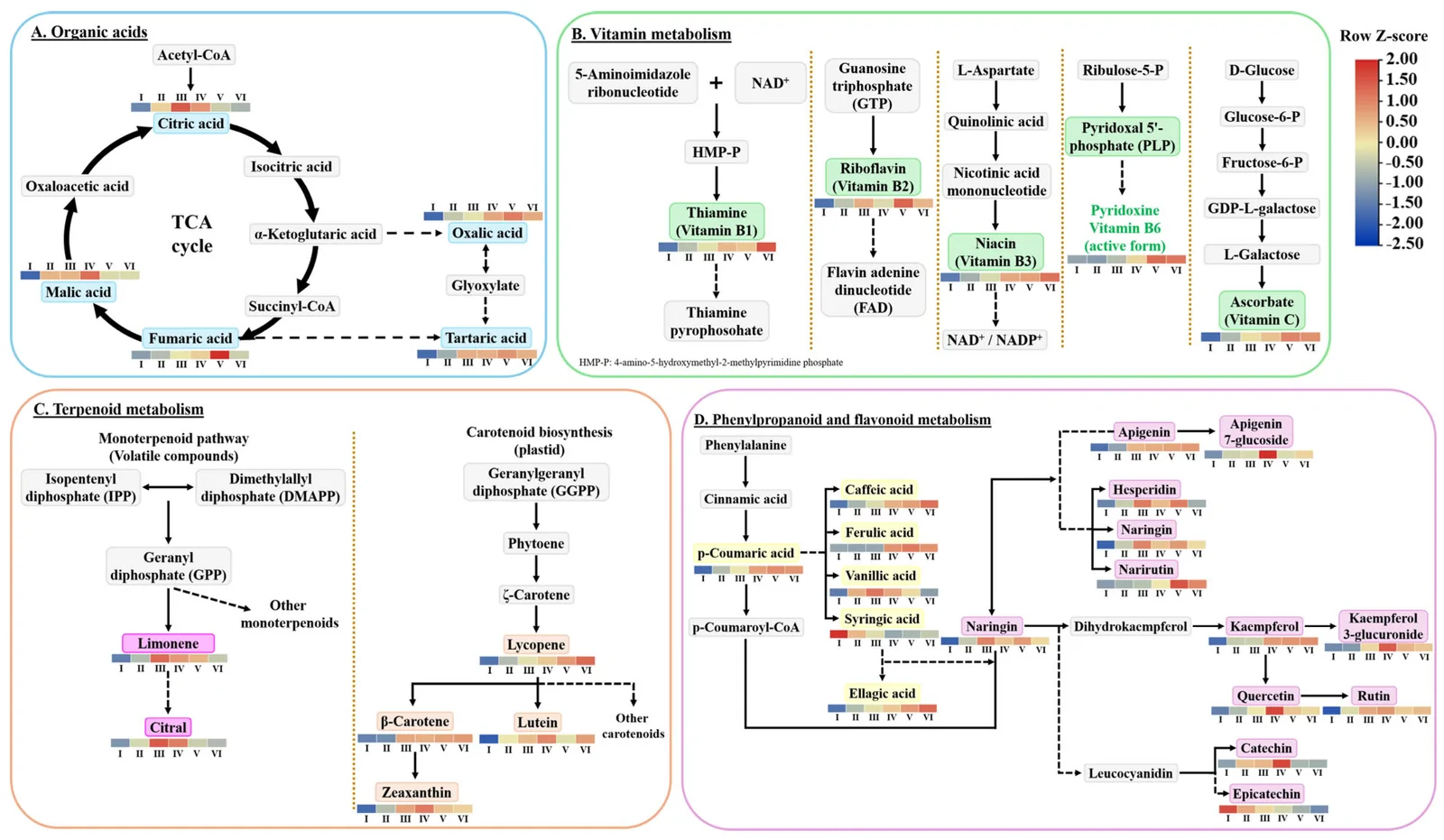
Heatmap showing the relative abundance patterns of carotenoids (beige boxes), flavonoids (pink boxes), phenolic acids (yellow boxes), organic acids (blue boxes), vitamins (green boxes), and volatile compounds (purple boxes) identified in orange peel during six fruit developmental stages. Gray boxes represent metabolic intermediates and precursors. Metabolite abundances were standardized by row-wise *Z*-score transformation, with red and blue representing relatively higher and lower metabolite abundances, respectively. Solid arrows indicate direct metabolic conversions, whereas dashed arrows indicate indirect or alternative metabolic connections.

The relative changes in phenolic acids, flavonoids, carotenoids, organic acids, vitamins and aroma-volatile compounds identified in orange peel throughout fruit development are presented in Figure 4. Because the heatmap uses row-based *Z*-score standardization for metabolites, the color intensities represent the relative variation in each compound across the six stages rather than its absolute concentration. This approach enabled the developmental patterns of different metabolite classes to be evaluated together. In general, organic acids reached their highest relative levels during the mid-growth period, largely reflecting the pattern observed for citric acid. Among the organic acids, citric and malic acids showed higher relative levels during the mid-growth period, whereas oxalic and tartaric acids showed relatively higher levels during late growth stages. Water-soluble vitamins such as thiamin, niacin, pyridoxine, and ascorbic acid generally showed higher relative levels during later stages. These changes are consistent with studies reporting that organic acid metabolism in citrus fruits is reorganized throughout development and that ascorbic acid accumulation within the peel of Valencia oranges is associated with development and color change [55,60].

Phenolic compounds, flavonoids, carotenoids and volatile compounds showed distinct accumulation patterns across development stages (Figure 4). Hou et al. [11] reported that phenolic acids in citrus fruits are synthesized through the shikimate and phenylpropanoid pathways, with the pathway from phenylalanine leading to cinnamic acid, *p*-coumaric acid and subsequently to hydroxycinnamic acid derivatives. Wang et al. [7] reported that cinnamic acid, formed by *phenylalanine ammonia lyase* (PAL), is directed toward phenylpropanoid and flavonoid metabolism through the actions of *cinnamate 4-monooxygenase* (C4H) and *4-coumarate-CoA ligase* (4CL), while flavonoid precursors such as naringenin are formed through the actions of *chalcone synthase* (CHS) and *chalcone isomerase* (CHI). This shared biosynthetic origin makes the joint assessment of phenolic acid and flavonoid profiles particularly relevant in the present study. Carotenoids are a large family of isoprenoid compounds biosynthesized through the tail-to-tail linkage of two geranylgeranyl diphosphate (GGPP) molecules [36]. Generally, carotenoids are divided into two groups: carotenes, which consist of a hydrocarbon backbone, and xanthophylls, which contain oxygen atoms in their structure. In the present study, many phenolic and flavonoid compounds reached higher relative levels during the early or mid-developmental stages, whereas *β*-carotene and lycopene reached their highest relative levels during the later stages, and limonene and citral reached their highest relative levels during the mid-stages. These results show that different metabolite groups in orange peel do not undergo simultaneous changes during fruit development [7,24,36] but instead exhibit metabolite-class- and compound-specific changes.

Kyoto Encyclopedia of Genes and Genomes (KEGG) pathway mapping further contextualized these developmental changes by linking the detected metabolites to several interconnected metabolic pathways (Figure 4). Phenolic acids and flavonoids were associated primarily with the phenylpropanoid and flavonoid pathways, whereas carotenoids were associated with the carotenoid biosynthesis pathway. The volatile terpenoids were mainly related to monoterpenoid metabolism. Organic acids were represented in central metabolic pathways, including the citrate cycle and glyoxylate/dicarboxylate metabolism, while the detected water-soluble vitamins were associated with their respective vitamin metabolic pathways. These pathway associations indicate that the developmental changes observed in orange peel involved coordinated shifts across secondary metabolism, organic-acid metabolism, and vitamin-related metabolism.

### 3.5. Mineral Composition of Orange Peels

The mineral composition of orange peel also showed significant changes (Table 7). Among the macro-elements, Ca and K were present at higher concentrations at all stages, though their concentrations decreased after Stage II and did not change at later stages. An increase was observed for Ca concentration from Stage V to Stage VI but was not significant. Calcium is an important element for its role in the formation of strong bones [6] and citrus fruits are valuable sources of calcium. Potassium plays significant roles in regulating water and electrolyte balance, and acid-base balance in humans [63]. Plant-derived Ca reported to be well absorbed by the human digestive system [64]. Though P is a macronutrient, its concentration was very low in peel samples. Among the eight micronutrients detected in orange peel, Mg had the highest concentration. As with K and Ca concentrations, Mg content also showed a consistent decrease from Stage I to Stage VI, falling from 0.740 mg g^−1^ in Stage I to 0.005 mg g^−1^ in Stage VI. Among the other microelements, Fe, Zn and Na were detected only in Stage I, at levels of 0.020, 0.003 and 0.016 mg g^−1^, respectively, while they remained below the detection limit at the subsequent sampling times. Mn was detected only in Stages I and II, and its concentration dropped below the detection limit at later stages. Cu, P, Se and Si remained below the analytical detection limits for all stages. These results indicate that the concentrations of mineral elements were generally higher at early stages of fruit development. Sharma et al. [12] observed that three different sweet orange varieties showed significant changes in macro- and micronutrient levels during fruit development, with Ca, Mg and Zn decreasing until mid-development and increasing at later stages, whereas P, K and Fe decreased continuously during fruit development. The results indicated that mineral elements do not undergo unisonous changes but rather exhibit mineral-specific changes. Peels of different *Citrus* species generally contained higher mineral concentrations than pulp, particularly for Ca, Mg, K, Fe, Zn and Mn [6]. When considered together with the increase in fruit size and peel mass, the decrease in mineral concentrations at later stages suggests that the observed changes cannot be explained solely by differences in mineral uptake; and tissue growth and redistribution of elements may have also contributed to these changes.

### 3.6. Antibacterial Activity

**Figure 5:**
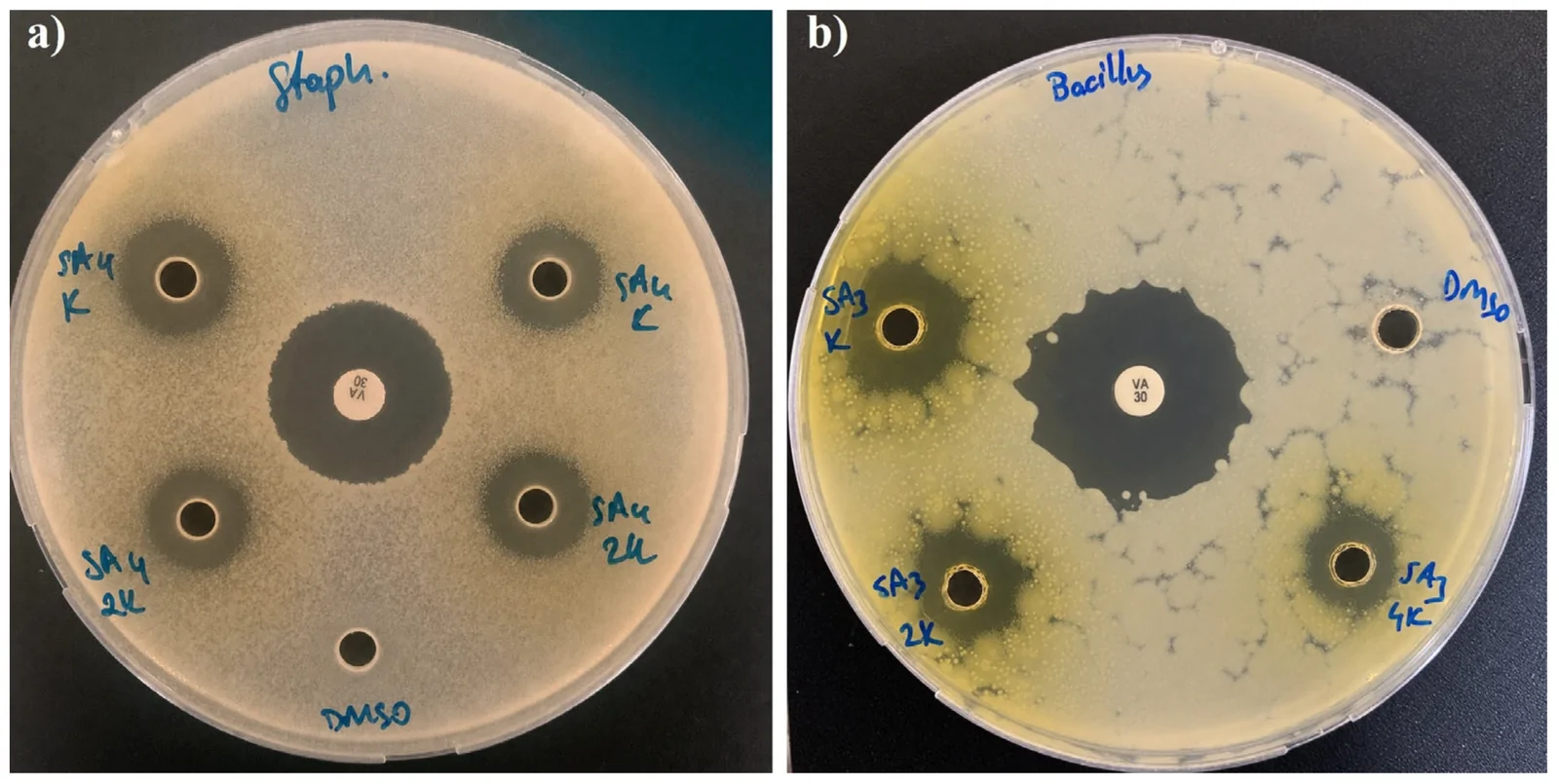
Agar well diffusion assay showing the antibacterial activity of orange peel extracts against (**a**) *S. aureus* and (**b**) *B. cereus*. Vancomycin (30 µg) and DMSO were used as positive and negative controls, respectively.

The antibacterial activity of methanolic orange peel extracts varied considerably depending on both the stage of fruit development and the microorganisms tested (Table 8). *B. cereus* was the only bacterium that showed growth inhibition against orange peel extracts from different stages, whereas antibacterial activity against *E. coli*, *E. faecalis* and *S. aureus* varied depending on developmental stages. In the agar well diffusion test, the largest inhibition zone was measured at Stage II (27.67 mm) for *E. faecalis* and at Stage IV (24.67 mm) for *E. coli*, both at an extract concentration of 500 mg mL^−1^. Overall, the decrease in inhibition zone diameters with decreasing peel extract concentration indicated that the observed antibacterial effect was concentration-dependent. The distinct inhibition zones formed by the extracts in the agar well diffusion assay are shown in Figure 5 for *S. aureus* and *B. cereus*. However, antibacterial activity observed in different stages cannot directly be linked to the total phenolics and flavonoid content. For example, while no inhibition of *E. coli* was observed at Stage II, when total phenolics and flavonoid content were highest, an inhibition zone of 24.67 mm was obtained against the same bacterium at Stage IV, when total phenolics and flavonoid content were significantly lower. This suggests that antibacterial efficacy may be related to the specific metabolite composition of the extract and possible synergistic interactions between compounds, rather than to total phenolics and flavonoid content alone. Figure 4 also shows that both phenolic and flavonoid compounds occurred at relatively high levels during Stages IV–V, supporting this hypothesis. Indeed, Table 3 shows that hesperidin, naringin, narirutin, quercetin, rutin, kaempferol and various phenolic acids exhibit different accumulation profiles during fruit development. Notably, the Stage IV extract, which produced a 24.67 mm inhibition zone against *E. coli*, contained substantially higher levels of several phenolic compounds than the Stage II extract, which showed no inhibition against this bacterium. For example, ferulic acid increased from 2691.7 to 5218.0 mg kg^−1^, quercetin from 487.6 to 1098.6 mg kg^−1^, narirutin from 1524.3 to 2190.8 mg kg^−1^ and kaempferol from 154.7 to 261.7 mg kg^−1^ between Stages II and IV (Table 3). Ferulic acid and quercetin have previously been reported to exhibit antibacterial activity against *E. coli*, with reported effects involving alterations in bacterial membrane integrity and permeability [65,66]. These observations support several phenolic compounds as plausible contributors to the stronger antibacterial response observed at Stage IV. Flavonoids are a large group of polyphenolic compounds that can inhibit DNA and RNA metabolism and synthesis in bacteria [67]. The antibacterial activity of citrus peel extracts has previously been linked to their flavonoid composition, and flavonoids have been reported to affect membrane integrity and permeability as well as cellular metabolism [8,68]. But total flavonoid content, along with phenolic content did not sufficiently explain the results observed in Table 8.

In addition to phenolic compounds, other volatile compounds may also have contributed to the observed antibacterial effects. The fact that other volatile compounds such as *α*-pinene, geraniol, linalool and myrcene were also detected at Stage III, when limonene (27.22 mg kg^−1^) and citral (0.33 mg kg^−1^) reached their highest levels, may be responsible for the observed inhibition of both *E. coli* and *E. faecalis* (Table 6; Table 8). The potential antibacterial activity of limonene and citral is supported by previous studies showing that limonene can disrupt membrane integrity and increase cellular leakage in *E. coli*, whereas citral has been reported to cause damage to both the outer and cytoplasmic membranes of *E. coli* [69,70]. Previous studies have also reported that volatile components of citrus peel, particularly terpenoids, contribute to antibacterial activity [71]. The MIC and MBC results further supported the stage- and microorganism-dependent nature of antibacterial activity. Overall, these findings suggest that the antibacterial potential of orange peel is not constant throughout development and that antibacterial efficacy may be related to the metabolite profile, which changes with development, rather than solely to total phenolics and flavonoid levels. Therefore, when evaluating orange peel as a natural source of antibacterial compounds, it is important to consider the harvest time and developmental stage in relation to the target microorganism.

### 3.7. Multivariate Analysis of Metabolite Profiles

**Figure 6:**
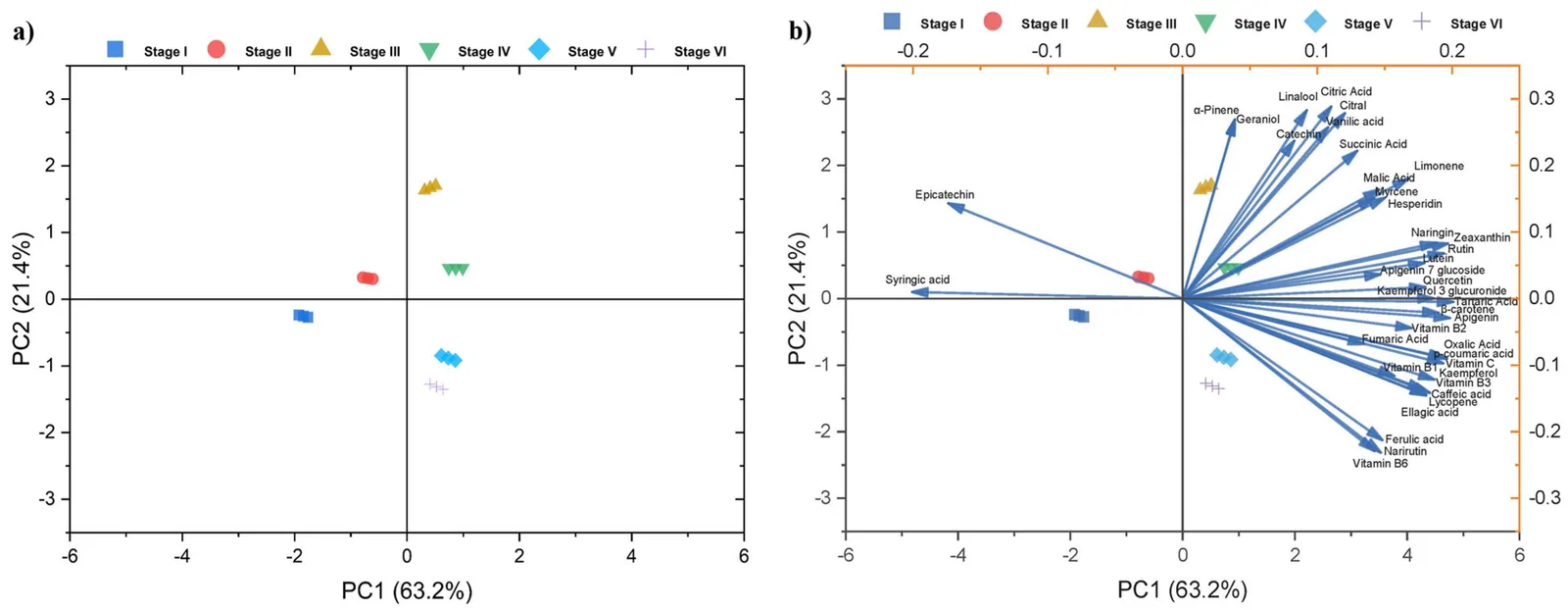
Principal component analysis (PCA) of orange peel metabolite profiles at six developmental stages. (**a**) Score plot and (**b**) biplot.

PCA, performed to collectively evaluate the identified metabolites, revealed a distinct multivariate distribution of the samples according to developmental stage (Figure 6). A total of 38 metabolites were included in the analysis, with three biological replicates from each developmental stage treated as separate observations. The first principal component (PC1) explained 63.24% of the total variance, while the second principal component (PC2) explained 21.40%; thus, the first two components together accounted for 84.63% of the total variance. The high cumulative proportion of explained variance indicates that developmental differences in metabolite composition can be largely represented in the two-dimensional PCA space. In the PCA score distribution, Stage I and Stage II samples were located in the negative PC1 region, whereas Stage III and Stage IV samples were positioned in the positive PC1 region. Although Stage V and Stage VI samples also fell within the positive PC1 region, they were distinguished from Stages III and IV by their lower scores along PC2 (Figure 6a). The close clustering of the biological replicates from each developmental stage indicates that the overall metabolite profiles of the samples within the same stage were similar, whereas the distribution across stages revealed changes in metabolite composition throughout development. The separation of Stage III, characterized by high positive PC2 scores, from Stage VI, characterized by distinctly negative PC2 scores, demonstrates that the metabolic differences between the intermediate and advanced developmental stages were strongly represented by the second principal component.

Biplot loadings demonstrated that this separation did not stem from a single metabolite class but rather reflected the joint contribution of compounds from different metabolite groups (Figure 6b). Among the loadings in the positive direction along PC1, tartaric acid, zeaxanthin, *p*-coumaric acid, rutin and oxalic acid were particularly prominent, whereas syringic acid and epicatechin contributed to the negative direction. Citric acid, geraniol, vanillic acid, catechin, and succinic acid exhibited more pronounced loadings in the positive direction of PC2, whereas ferulic acid, narirutin, and vitamin B_6_ contributed to the negative direction. This distribution suggests that the distinction between developmental stages cannot be explained solely by changes in total phenolic or flavonoid content; rather, the co-varying profiles of organic acids, phenolic compounds, flavonoids, carotenoids, vitamins, and volatile compounds contributed to the overall metabolic differentiation.

These findings are consistent with the different developmental trends observed for individual metabolites in the present study. Indeed, studies on orange peel have reported that phenolic compounds and flavonoids do not exhibit a uniform pattern throughout development; while some compounds are present at higher levels during the early stages, others increase as ripening progresses [7]. Similarly, carotenoid accumulation has been shown to accelerate particularly during the coloration phase, with the carotenoid composition undergoing changes throughout development [39]. Significant developmental-stage-dependent changes in volatile compounds have also been reported, and terpenoids such as *d*-limonene and linalool have been shown to exhibit distinct accumulation profiles throughout development [13]. Therefore, the current PCA results support the view that changes observed separately across different metabolite classes, when evaluated together, form a broader metabolic profile characteristic of each developmental stage.

**Figure 7:**
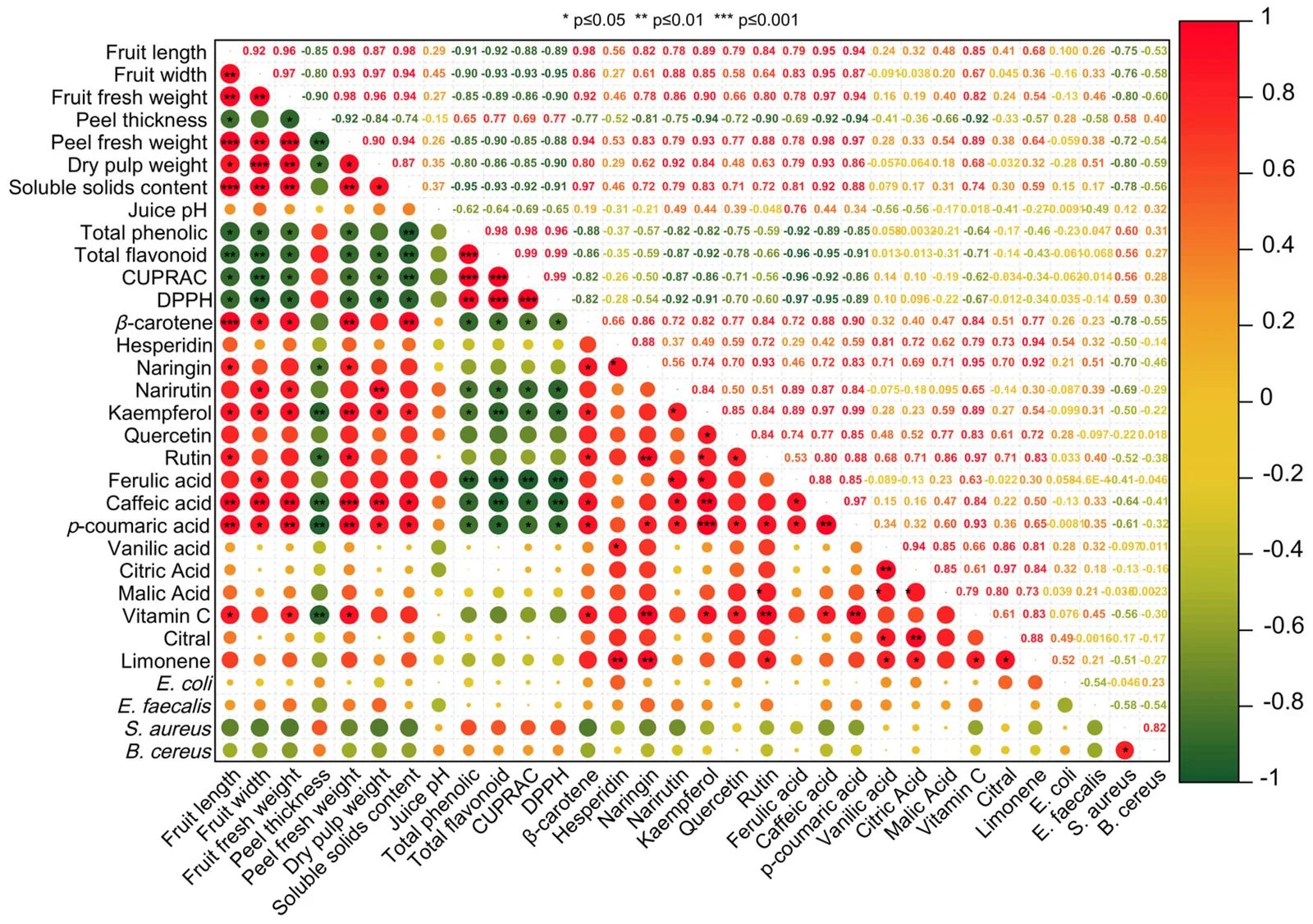
Correlation matrix showing the relationships among fruit characteristics, biochemical parameters, selected metabolites, and antibacterial activities of orange peel extracts across the six developmental stages. Red and green colors indicate positive and negative correlations, respectively.

Correlation analysis across the six developmental stages revealed significant stage-dependent relationships among fruit development, biochemical properties, selected metabolites, and antibacterial activity at an extract concentration of 500 mg mL^−1^ (Figure 7). Strong positive correlations were observed among fruit length, fruit width, fruit fresh weight, peel fresh weight, and dry pulp weight, whereas peel thickness was negatively associated with most of these traits. The relationships between fruit length and fruit width (*r* = 0.92), fruit width and fruit fresh weight (*r* = 0.97), fruit fresh weight and dry pulp weight (*r* = 0.96) and fruit length and soluble solids content (*r* = 0.98) were particularly noteworthy. In contrast, peel thickness showed negative correlations with fruit length (*r* = −0.85), fruit width (*r* = −0.80) and fruit fresh weight (*r* = −0.90). These relationships are consistent with fruit expansion and dry matter accumulation in the internal tissues accompanied by a relative decrease in peel thickness. While ripening in citrus fruits is characterized by simultaneous changes in size, soluble solids, acidity, color, and metabolite composition, the development of peel and pulp has also been reported to be not entirely synchronous [21]. Regarding antioxidant properties, the most prominent pattern was the very strong positive intercorrelations among total phenolic content, total flavonoid content with CUPRAC and DPPH values. The correlation coefficient between total phenolics and total flavonoid content was *r* = 0.98, while the correlation coefficients between total phenolic content with CUPRAC and DPPH were *r* = 0.99 and *r* = 0.99, respectively; a very strong relationship (*r* = 0.99) was also observed between CUPRAC and DPPH. These results indicate that changes in the total phenolics and flavonoid pools during development closely paralleled changes in antioxidant capacity. Similarly, a strong relationship (*r*^2^ = 0.95) between total phenolics content and FRAP has been reported for *C. sinensis* peel extracts [4], while in navel oranges, antioxidant capacity was shown to be significantly correlated with total phenolics, flavonoids and various phenolic acids [11]. However, the relationships of certain individual metabolites with antioxidant parameters exhibited developmental patterns distinct from the general trend observed for total phenolic and flavonoid contents. *β*-carotene showed strong negative correlations with total phenolics (*r* = −0.88), total flavonoid content (*r* = −0.86), CUPRAC (*r* = −0.82) and DPPH (*r* = −0.82). These relationships should not be interpreted as indicating that *β*-carotene negatively affects antioxidant capacity; rather, they should be viewed because of the two groups of variables change in opposite directions during fruit development. Indeed, carotenoid accumulation has been reported to accelerate as coloration progresses in citrus peel, with *β*-carotene and other carotenoids undergoing marked changes during ripening [36,39]. Distinct relationships were also observed among individual phenolic and flavonoid compounds. Hesperidin, naringin, narirutin, kaempferol, quercetin, and rutin mostly showed positive correlations with one another; strong positive relationships were observed among the phenolic acids, particularly between ferulic, caffeic and *p*-coumaric acids. These results suggest that these compounds exhibit similar accumulation profiles throughout fruit development. Studies on orange peel have reported that phenolic compounds are generally present at higher levels during the young and semi-mature stages and decrease at commercial maturity; moreover, the developmental profiles of individual compounds may differ from one another [7]. Hou et al. [11] also reported that phenolic acids such as ferulic, caffeic, and *p*-coumaric acids can exhibit significant correlations with antioxidant capacity.

Among the organic acids, citric and malic acids showed strong positive correlations, while the relationship between citral and limonene was also significant among the volatile compounds. This suggests that the profiles of organic acids and volatile compounds do not change independently during fruit development but instead exhibit stage-specific patterns. Previous studies have reported that the volatile components of orange peel vary significantly according to developmental stage and that limonene is one of the major volatile compounds throughout development [13].

The relationships between antibacterial activity and metabolites did not reveal a significant correlation (Figure 7). Moderate positive correlations were observed between the *E. coli* inhibition zone at 500 mg mL^−1^ and limonene (*r* = 0.52) and citral (*r* = 0.49), whereas a stronger positive correlation (*r* = 0.82) was found between the inhibition zones for *S. aureus* and *B. cereus*. In contrast, the relationships between total phenolics and flavonoid contents and bacterial inhibition were not uniform across the tested microorganisms. When considered together with the developmental stage-specific differences in antibacterial activity observed in the present study, these findings suggest that the antibacterial response cannot be explained solely by total phenolics or flavonoid content and that the composition of individual metabolites and microbial susceptibility should be considered together. Previous studies have reported that citrus peel extracts exhibit antibacterial activity against various Gram-negative and Gram-positive bacteria and that this activity may be related to the chemical composition of the extracts [8,61].

## 4. Conclusions

This study demonstrated that the chemical composition and biological properties of orange peels are affected by fruit development stage. All variables changed significantly between developmental stages and total phenolics and flavonoid content, as well as antioxidant activity, were generally higher at early stages of fruit development. However, UHPLC-MS/MS analyses revealed distinct accumulation profiles among individual metabolites throughout development. Hesperidin, ferulic acid and *β*-carotene emerged as prominent compounds within the flavonoid, phenolic acid and carotenoid groups, respectively; notably, carotenoid accumulation increased markedly with color development of peel tissues. Citric and malic acids were key components of the organic acid profile, while ascorbic acid was the major component of the water-soluble vitamins; limonene, the dominant aroma-volatile compound, reached its highest level during the third developmental stage. The concentrations of mineral elements, on the other hand, were generally higher during the early stages of development and dropped significantly as fruit and peel development progressed. Antibacterial activity varied depending on stage and the microorganism tested, while *B. cereus* was inhibited by extracts obtained from all development stages; the other bacteria showed more variable responses. Multivariate analysis further supported development-related changes in metabolite profiles, while correlation analysis revealed strong associations between fruit traits, biochemical parameters, and selected metabolites. The lack of direct correspondence between antibacterial activity and total phenolics or flavonoid content indicates that the composition of individual metabolites and potential interactions among the compounds should also be taken into account. These findings demonstrate that the chemical and biological properties of orange peel should not be evaluated solely on the basis of the total amount of bioactive compounds, and that dynamic changes specific to metabolite classes and to individual compounds occur during development. Furthermore, the fruit developmental stage is a significant factor determining the potential use of the peel. Therefore, selecting the developmental stage according to the target compound or biological property may be an alternative strategy for utilizing orange peel in value-added applications.

## Figures and Tables

**Table 1:** Monthly mean temperature (°C), total precipitation (mm) and relative humidity (%) in 2024 and their long-term averages (1972–2024) for Serik district, Antalya province, Türkiye.

Months	Temperature (°C)		Precipitation (mm)		Humidity (%)	
	2024	1972–2024	2024	1972–2024	2024	1972–2024
January	13.1	11.1	177.5	264.5	66.1	64.4
February	13.5	12.1	96.9	87.7	65.2	60.4
March	15.3	14.4	14.1	81.5	57.2	59.6
April	21.0	17.6	15.0	33.2	57.1	62.1
May	21.8	21.8	18.9	39.9	60.1	62.4
June	29.9	26.4	0.2	18.1	53.3	58.5
July	31.6	30.2	0.0	1.8	47.2	51.9
August	30.4	29.9	0.1	5.5	55.7	59.1
September	26.9	27.2	0.0	25.2	55.0	54.4
October	22.9	22.6	7.9	66.6	45.3	52.7
November	17.3	17.6	216.7	136.3	49.9	55.6
December	13.2	13.2	238.7	217.8	73.2	65.8
Total	-	-	786.0	978.1	-	-
Mean	21.4	20.3	-	-	57.1	58.9

**Table 2:** Sampling dates and phenological characteristics of Valencia orange fruits at six developmental stages.

Developmental Stage	Phenological Characteristics	Days After Flowering (DAF)	Sampling Date
Stage I	Early fruit development; small, immature green fruit	120	27 August 2024
Stage II	Fruit enlargement begins; fruit remains green	150	26 September 2024
Stage III	Active fruit enlargement; early changes in peel coloration are observed	180	26 October 2024
Stage IV	Fruit enlargement continues, and the onset of peel coloration becomes evident	210	25 November 2024
Stage V	Fruit approaches physiological maturity, and peel coloration becomes more pronounced	230	15 December 2024
Stage VI	Fruit reaches harvest maturity	250	4 January 2025

**Table 3:** Changes in the concentrations of phenolic acid, flavonoid and carotenoid components in orange peel during six fruit developmental stages.

Class	Compounds (mg kg^−1^)	Fruit Development Stage (Orange Peel)					
		Stage I	Stage II	Stage III	Stage IV	Stage V	Stage VI
**Carotenoids**	**Carotenes**						
	*β*-carotene	3748.2 ± 11.0 ^f^	4025.9 ± 12.7 ^e^	8275.9 ± 15.0 ^c^	8148.2 ± 14.1 ^d^	8372.3 ± 11.3 ^b^	8596.4 ± 10.4 ^a^
	Lycopene	127.3 ± 3.8 ^g^	154.9 ± 2.3 ^e^	168.7 ± 3.5 ^d^	182.6 ± 4.6 ^c^	196.4 ± 5.7 ^b^	210.2 ± 5.2 ^a^
	**Xanthophylls**						
	Astaxanthin	N.D.	N.D.	N.D.	N.D.	N.D.	N.D.
	Lutein	351.2 ± 2.8 ^f^	796.7 ± 4.5 ^d^	920.6 ± 6.3 ^c^	1044.6 ± 7.4 ^a^	768.6 ± 5.1 ^e^	992.5 ± 6.4 ^b^
	Zeaxanthin	342.8 ± 2.9 ^f^	421.4 ± 4.5 ^e^	510.8 ± 4.2 ^b^	541.1 ± 3.4 ^a^	489.4 ± 3.5 ^c^	478.7 ± 4.7 ^d^
**Flavonoids**	**Anthocyanins**						
	Cyanidin	N.D.	N.D.	N.D.	N.D.	N.D.	N.D.
	Delphinidin	N.D.	N.D.	N.D.	N.D.	N.D.	N.D.
	**Flavones**						
	Apigenin	226.8 ± 2.6 ^f^	277.5 ± 2.7 ^e^	397.7 ± 3.5 ^d^	405.8 ± 2.6 ^c^	414.0 ± 2.8 ^b^	422.1 ± 3.9 ^a^
	Apigenin 7 glucoside	118.6 ± 1.3 ^e^	177.8 ± 1.2 ^d^	185.4 ± 1.4 ^c^	337.0 ± 3.1 ^a^	186.5 ± 1.8 ^c^	219.1 ± 2.6 ^b^
	**Flavanones**						
	Hesperidin	27,728.5 ± 13.9 ^f^	35,097.8 ± 19.3 ^d^	47,161.0 ± 25.7 ^a^	42,124.2 ± 22.3 ^c^	47,087.3 ± 26.5 ^b^	32,050.5 ± 18.2 ^e^
	Naringin	3741.1 ± 12.5 ^f^	5038.7 ± 14.1 ^e^	6428.5 ± 14.3 ^a^	5818.4 ± 13.4 ^c^	6208.3 ± 12.8 ^b^	5598.1 ± 11.9 ^d^
	Narirutin	1257.9 ± 7.5 ^f^	1524.3 ± 8.3 ^e^	1707.5 ± 9.4 ^d^	2190.8 ± 10.7 ^c^	3674.0 ± 13.4 ^a^	3157.2 ± 11.8 ^b^
	**Flavanols**						
	Catechin	371.4 ± 2.8 ^f^	485.6 ± 4.1 ^c^	492.8 ± 4.5 ^b^	584.1 ± 4.8 ^a^	393.0 ± 3.4 ^d^	385.9 ± 3.5 ^e^
	Epicatechin	284.7 ± 2.9 ^a^	272.4 ± 2.7 ^b^	266.2 ± 2.6 ^c^	260.1 ± 2.5 ^d^	253.9 ± 2.7 ^e^	247.7 ± 2.3 ^f^
	Epicatechin gallate	N.D.	N.D.	N.D.	N.D.	N.D.	N.D.
	Epigallocatechin	N.D.	N.D.	N.D.	N.D.	N.D.	N.D.
	Epigallocatechin gallate	N.D.	N.D.	N.D.	N.D.	N.D.	N.D.
	Gallocatechin	N.D.	N.D.	N.D.	N.D.	N.D.	N.D.
	Kaempferol	47.7 ± 0.3 ^f^	154.7 ± 1.2 ^e^	158.2 ± 1.3 ^d^	261.7 ± 2.4 ^c^	265.3 ± 2.1 ^b^	268.8 ± 2.5 ^a^
	Kaempferol 3 glucuronide	5.3 ± 0.03 ^f^	5.8 ± 0.02 ^e^	9.3 ± 0.07 ^d^	12.8 ± 0.08 ^a^	10.3 ± 0.07 ^b^	9.8 ± 0.09 ^c^
	Quercetin	276.6 ± 2.7 ^f^	487.6 ± 4.6 ^e^	643.1 ± 5.1 ^d^	1098.6 ± 8.7 ^a^	754.1 ± 6.3 ^b^	709.6 ± 5.7 ^c^
	Quercetin 3 glucuronide	N.D.	N.D.	N.D.	N.D.	N.D.	N.D.
	Quercetin 3 sulfate	N.D.	N.D.	N.D.	N.D.	N.D.	N.D.
	Rutin	284.6 ± 2.5 ^f^	779.7 ± 5.1 ^e^	1027.2 ± 6.8 ^b^	1074.7 ± 7.2 ^a^	922.3 ± 6.9 ^d^	969.8 ± 7.1 ^c^
**Phenolic Acids**	**Hydroxycinnamic acids**						
	Ferulic acid	2765.3 ± 10.5 ^d^	2691.7 ± 9.3 ^e^	2754.8 ± 7.5 ^d^	5218.0 ± 13.3 ^c^	5581.2 ± 13.5 ^a^	5244.3 ± 14.8 ^b^
	Rosmarinic acid	N.D.	N.D.	N.D.	N.D.	N.D.	N.D.
	Caffeic acid	2524.1 ± 10.7 ^f^	2781.2 ± 11.2 ^e^	2933.9 ± 9.4 ^d^	3186.5 ± 13.8 ^c^	3239.2 ± 12.7 ^b^	3391.8 ± 11.3 ^a^
	Caftaric acid	N.D.	N.D.	N.D.	N.D.	N.D.	N.D.
	*p*-coumaric acid	732.1 ± 5.2 ^f^	1078.9 ± 5.5 ^e^	1244.4 ± 7.4 ^d^	1489.9 ± 8.6 ^c^	1545.4 ± 9.4 ^a^	1523.9 ± 9.6 ^b^
	**Hydroxycinnamate esters**						
	Chlorogenic acid	N.D.	N.D.	N.D.	N.D.	N.D.	N.D.
	**Hydroxybenzoic acids**						
	Gallic acid	N.D.	N.D.	N.D.	N.D.	N.D.	N.D.
	Syringic acid	363.2 ± 2.8 ^a^	301.1 ± 2.4 ^b^	270.0 ± 2.4 ^c^	238.9 ± 1.4 ^f^	247.8 ± 1.5 ^e^	257.8 ± 1.3 ^d^
	**Hydrolysable tannins**						
	Ellagic acid	278.5 ± 1.4 ^f^	290.9 ± 1.7 ^e^	297.0 ± 2.1 ^d^	303.2 ± 2.5 ^c^	309.4 ± 2.4 ^b^	315.5 ± 2.3 ^a^
	**Hydroxybenzoates**						
	Vanillic acid	1379.7 ± 8.5 ^f^	1984.6 ± 6.8 c	2163.0 ± 7.3 ^a^	2010.5 ± 8.9 ^b^	1858.1 ± 7.2 ^d^	1505.6 ± 6.3 ^e^
**Class compositions**							
Carotenoids		4569.5	5398.9	9876	9916.5	9826.7	10,277.8
Flavonoids		34,343.2	44,301.9	58,476.9	54,168.2	60,169	44,038.6
Phenolic Acids		8042.9	9128.4	9663.1	12,447	12,781.1	12,238.9
**Total**		46,955.6	58,829.2	78,016.0	76,531.7	82,776.8	66,555.3

Different lowercase letters indicate significant differences among developmental stages according to Duncan’s multiple range test (*p* ≤ 0.05). (N.D. = not detected; LOD = 5 mg kg^−1^).

**Table 4:** Concentrations of organic acids in orange peel at six sampling times during fruit development.

Organic Acids (mg kg^−1^)	Fruit Development Stage (Orange Peel)					
	Stage I	Stage II	Stage III	Stage IV	Stage V	Stage VI
Acetic Acid	N.D.	N.D.	N.D.	N.D.	N.D.	N.D.
Citric Acid	37,284.7 ± 580.4 ^f^	47,290.2 ± 546.7 ^c^	55,266.4 ± 601.2 ^a^	51,238.1 ± 574.1 ^b^	43,231.8 ± 490.2 ^d^	41,227.5 ± 516.4 ^e^
Fumaric Acid	16.0 ± 2.7 ^c^	18.2 ± 1.9 ^bc^	21.2 ± 2.3 ^bc^	23.1 ± 2.8 ^b^	32.1 ± 3.6 ^a^	19.6 ± 2.9 ^bc^
Lactic Acid	N.D.	N.D.	N.D.	N.D.	N.D.	N.D.
Malic Acid	8908.0 ± 221.4 ^d^	15,130.1 ± 317.4 ^b^	15,156.1 ± 320.5 ^b^	17,119.1 ± 390.8 ^a^	13,089.5 ± 346.7 ^c^	13,080.4 ± 317.4 ^c^
Oxalic Acid	649.3 ± 27.5 ^e^	1272.6 ± 45.7 ^d^	1448.5 ± 48.3 ^c^	1846.8 ± 43.2 ^b^	2043.5 ± 53.4 ^a^	1826.9 ± 47.2 ^b^
Propionic Acid	N.D.	N.D.	N.D.	N.D.	N.D.	N.D.
Succinic Acid	40.2 ± 3.4 ^c^	53.0 ± 4.5 ^b^	79.6 ± 6.4 ^a^	61.2 ± 5.7 ^b^	53.9 ± 4.8 ^b^	56.4 ± 4.3 ^b^
Tartaric Acid	900.7 ± 34.4 ^d^	1514.4 ± 53.3 ^c^	2496.3 ± 57.1 ^b^	2485.7 ± 54.6 ^b^	2685.5 ± 56.3 ^a^	2479.7 ± 55.3 ^b^
Total	47,798.9	65,278.5	74,468.1	72,774.0	61,136.3	58,690.5

Different lowercase letters indicate significant differences among developmental stages according to Duncan’s multiple range test (*p* ≤ 0.05). (N.D. = not detected; LOD = 10 mg kg^−1^).

**Table 5:** Changes in the concentrations of water-soluble vitamins in orange peel during six fruit developmental stages.

Water-Soluble Vitamins (mg kg^−1^)	Fruit Development Stage (Orange Peel)					
	Stage I	Stage II	Stage III	Stage IV	Stage V	Stage VI
Thiamin (Vitamin B_1_)	9.73 ± 0.85 ^c^	11.24 ± 1.01 ^bc^	11.87 ± 1.06 ^b^	12.85 ± 0.97 ^ab^	12.62 ± 1.24 ^ab^	14.42 ± 1.13 ^a^
Riboflavin (Vitamin B_2_)	2.34 ± 0.04 ^d^	2.44 ± 0.02 ^c^	2.55 ± 0.02 ^b^	2.47 ± 0.04 ^c^	2.62 ± 0.03 ^a^	2.54 ± 0.03 ^b^
Niacin (Vitamin B_3_)	6.41 ± 0.53 ^c^	7.85 ± 0.74 ^b^	8.92 ± 0.08 ^b^	10.81 ± 1.01 ^a^	10.97 ± 0.98 ^a^	11.92 ± 0.93 ^a^
Pantothenic acid (Vitamin B_5_)	N.D.	N.D.	N.D.	N.D.	N.D.	N.D.
Pyridoxine (Vitamin B_6_)	1.31 ± 0.01 ^d^	1.29 ± 0.02 ^d^	1.37 ± 0.02 ^c^	1.52 ± 0.01 ^b^	1.71 ± 0.02 ^a^	1.69 ± 0.02 ^a^
Biotin (Vitamin B_7_)	N.D.	N.D.	N.D.	N.D.	N.D.	N.D.
Folic acid (Vitamin B_9_)	N.D.	N.D.	N.D.	N.D.	N.D.	N.D.
Cobalamin (Vitamin B_12_)	N.D.	N.D.	N.D.	N.D.	N.D.	N.D.
Ascorbic acid (Vitamin C)	1078.5 ± 10.2 ^e^	1240.7 ± 11.7 ^d^	1360.7 ± 12.7 ^c^	1407.4 ± 14.3 ^b^	1508.3 ± 13.6 ^a^	1487.6 ± 15.4 ^a^

Different lowercase letters indicate significant differences among developmental stages according to Duncan’s multiple range test (*p* ≤ 0.05). (N.D. = not detected; LOD = 1 mg kg^−1^).

**Table 6:** Changes in the concentrations of aroma-volatile compounds of orange peel during six fruit developmental stages.

Aroma-Volatile (mg kg^−1^)	Fruit Development Stage (Orange Peel)					
	Stage I	Stage II	Stage III	Stage IV	Stage V	Stage VI
*α*-Pinene	N.D.	N.D.	0.11 ± 0.001 ^a^	N.D.	N.D.	N.D.
Citral	0.12 ± 0.001 ^e^	0.19 ± 0.002 ^c^	0.33 ± 0.01 ^a^	0.30 ± 0.01 ^b^	0.18 ± 0.002 ^c^	0.16 ± 0.001 ^d^
Geraniol	N.D.	N.D.	0.14 ± 0.002 ^a^	N.D.	N.D.	N.D.
Limonene	7.71 ± 0.12 ^e^	13.43 ± 0.48 ^d^	27.22 ± 1.15 ^a^	23.74 ± 1.13 ^b^	21.77 ± 1.08 ^c^	14.71 ± 0.53 ^d^
Linalool	N.D.	N.D.	0.14 ± 0.002 ^a^	0.12 ± 0.001 ^a^	N.D.	N.D.
Myrcene	N.D.	N.D.	0.11 ± 0.001 ^b^	0.12 ± 0.001 ^a^	0.11 ± 0.001 ^b^	N.D.

Different lowercase letters indicate significant differences among developmental stages according to Duncan’s multiple range test (*p* ≤ 0.05). (N.D. = not detected; LOD = 0.1 mg kg^−1^).

**Table 7:** Changes in macro- and microelement concentrations in orange peel during six fruit developmental stages.

Mineral Composition (mg g^−1^)	Fruit Development Stage (Orange Peel)					
	Stage I	Stage II	Stage III	Stage IV	Stage V	Stage VI
Ca	8.094 ± 1.81 ^a^	5.346 ± 1.09 ^b^	1.923 ± 0.56 ^c^	0.226 ± 0.11 ^c^	0.276 ± 0.18 ^c^	0.646 ± 0.40 ^c^
Cu	<0.000008	<0.000008	<0.000008	<0.000008	<0.000008	<0.000008
Fe	0.02 ± 0.01 ^a^	<0.000005	<0.000005	<0.000005	<0.000005	<0.000005
K	7.880 ± 0.37 ^a^	4.988 ± 0.84 ^b^	0.390 ± 0.19 ^c^	0.458 ± 0.17 ^c^	0.375 ± 0.28 ^c^	0.100 ± 0.04 ^c^
Mg	0.740 ± 0.12 ^a^	0.390 ± 0.03 ^b^	0.160 ± 0.05 ^c^	0.020 ± 0.01 ^d^	0.013 ± 0.01 ^d^	0.005 ± 0.00 ^d^
Mn	0.003 ± 0.00 ^a^	0.001 ± 0.00 ^b^	<0.000002	<0.000002	<0.000002	<0.000002
Na	0.016 ± 0.01 ^a^	<0.00001	<0.00001	<0.00001	<0.00001	<0.00001
P	<0.000015	<0.000015	<0.000015	<0.000015	<0.000015	<0.000015
Se	<0.000025	<0.000025	<0.000025	<0.000025	<0.000025	<0.000025
Si	<0.000012	<0.000012	<0.000012	<0.000012	<0.000012	<0.000012
Zn	0.003 ± 0.00 ^a^	<0.000002	<0.000002	<0.000002	<0.000002	<0.000002

Different lowercase letters indicate significant differences among developmental stages according to Duncan’s multiple range test (*p* ≤ 0.05).

**Table 8:** Antibacterial activity of orange peel extracts obtained at six fruit developmental stages against selected Gram-positive and Gram-negative bacteria.

Fruit Development Stage (Orange Peel)	Concentration (mg mL^−1^)	*Escherichia coli* ATCC 25922	*Enterococcus faecalis* ATCC 29212	*Staphylococcus aureus* ATCC 29213	*Bacillus cereus* ATCC 11778
Stage 1	500	19.97 ± 0.05 mm	0	14.83 ± 0.26 mm	20.33 ± 0.52 mm
	250	14.67 ± 0.52 mm	0	13.00 ± 0.01 mm	17.67 ± 1.37 mm
	125	12.33 ± 0.52 mm	0	0	16.00 ± 0.89 mm
	MBC (mg mL^−1^)	62.5	N.I.	125	31.25
	MIC (mg mL^−1^)	62.5	N.I.	62.5	31.25
Stage 2	500	0	27.67 ± 0.52 mm	15.67 ± 0.52 mm	20.33 ± 0.52 mm
	250	0	16.67 ± 1.03 mm	12.33 ± 0.52 mm	19.00 ± 0.89 mm
	125	0	13.67 ± 0.52 mm	0	16.00 ± 0.89 mm
	MBC (mg mL^−1^)	N.I.	31.25	3.91	62.5
	MIC (mg mL^−1^)	N.I.	15.63	0.24	62.5
Stage 3	500	23.67 ± 0.52 mm	23.67 ± 1.37 mm	0	16.00 ± 0.89 mm
	250	19.83 ± 0.26 mm	18.00 ± 0.89 mm	0	12.00 ± 0.89 mm
	125	15.33 ± 0.52 mm	13.33 ± 1.37 mm	0	10.33 ± 0.52 mm
	MBC (mg mL^−1^)	62.5	1.95	N.I.	62.5
	MIC (mg mL^−1^)	62.5	0.24	N.I.	62.5
Stage 4	500	24.67 ± 0.52 mm	0	13.67 ± 0.52 mm	21.00 ± 0.89 mm
	250	19.67 ± 0.52 mm	0	11.50 ± 0.45 mm	14.67 ± 0.52 mm
	125	13.33 ± 1.03 mm	0	0	13.00 ± 0.89 mm
	MBC (mg mL^−1^)	62.5	N.I.	31.25	62.5
	MIC (mg mL^−1^)	62.5	N.I.	0.49	62.5
Stage 5	500	20.67 ± 1.03 mm	25.33 ± 0.52 mm	0	19.33 ± 1.03 mm
	250	17.00 ± 0.89 mm	19.00 ± 0.89 mm	0	15.33 ± 1.37 mm
	125	13.67 ± 0.52 mm	19.00 ± 0.89 mm	0	12.00 ± 0.89 mm
	MBC (mg mL^−1^)	62.5	62.5	N.I.	62.5
	MIC (mg mL^−1^)	62.5	62.5	N.I.	62.5
Stage 6	500	0	24.00 ± 0.89 mm	0	15.67 ± 1.03 mm
	250	0	17.00 ± 2.37 mm	0	0
	125	0	14.33 ± 2.25 mm	0	0
	MBC (mg mL^−1^)	N.I.	62.5	N.I.	250
	MIC (mg mL^−1^)	N.I.	62.5	N.I.	250
Gentamisin		26.42 ± 1.84 mm	N.I.	N.I.	N.I.
Vancomycin		N.I.	22.75 ± 6.97 mm	22.50 ± 0.97 mm	25.60 ± 1.38 mm
DMSO		N.I.	N.I.	N.I.	N.I.

N.I.: no inhibition; MIC: minimum inhibitory concentration; MBC: minimum bactericidal concentration. Values are presented as mean ± standard deviation.

## Data Availability

The original contributions presented in this study are included in the article. Further inquiries can be directed to the corresponding author.
